# Lipid Disorder and PIP2-Regulated Clustering of Syntaxin-1 JMD–TMD Regions Govern Membrane Fusion Competence

**DOI:** 10.3390/ijms27156673

**Published:** 2026-07-27

**Authors:** Dong An, Manfred Lindau

**Affiliations:** Department of Physiology and Biophysics, University of Miami Miller School of Medicine, Miami, FL 33136, USA; mxl2044@med.miami.edu

**Keywords:** syntaxin 1 isoforms, JMD-TMD clustering, lipid disorder, palmitoylation, PIP_2_ depletion, Stx1 JMD-PIP_2_ sequestration, membrane fusion, neurotransmitter release

## Abstract

Syntaxin-1 (Stx1), a core neuronal SNARE protein, regulates membrane docking and fusion during neurotransmitter release. Although Stx1 clustering is widely observed at presynaptic active zones and in neuroendocrine cells, how Stx1 isoforms and lipid interactions organize the plasma membrane prior to fusion remains unclear. Here, we performed MARTINI coarse-grained molecular dynamics (cgMD) simulations of 1–10 copies of the juxtamembrane and transmembrane domains (JMD–TMDs) of Stx1A and Stx1B embedded in plasma membrane models to investigate membrane lipid disorder, Stx1 JMD–TMD clustering dynamics, and regulation by Stx1 TMD palmitoylation and phosphatidylinositol 4,5-bisphosphate (PIP_2_). Stx1 JMD–TMD regions induced local lipid disorder associated with hydrophobic mismatch, with Stx1A producing stronger and more spatially extended perturbations than Stx1B. The magnitude of local lipid disorder remained largely independent of clustering and TMD palmitoylation, whereas palmitoylation spatially restricted and PIP_2_ depletion broadened the radial extent of membrane perturbation. During clustering, Stx1 JMD–TMDs progressively recruited and redistributed PIP_2_ into local membrane domains, resulting in ~7-fold local enrichment relative to the bulk membrane. Together, these findings support a model where hydrophobic mismatch primarily governs membrane disorder, whereas multivalent PIP_2_ recruitment and redistribution stabilize higher-order Stx1 oligomers, thereby promoting membrane environments permissive for SNARE-mediated fusion.

## 1. Introduction

Exocytosis, including neurotransmitter release, hormone secretion, and postsynaptic receptor trafficking, is mediated through coordinated membrane docking, priming, and membrane fusion events [1,2]. These processes are essential for maintaining neuronal communication, synaptic plasticity, and endocrine homeostasis [3]. In neurons, membrane fusion is primarily driven by the SNARE machinery, in which Syntaxin-1 (Stx1), a plasma membrane-anchored SNARE protein, interacts with SNAP25 and Synaptobrevin-2 (Syb2) to assemble the SNARE complex under regulation by Munc18 and Munc13 [4,5,6]. Because of its central role in organizing fusion sites, Stx1 contributes to establishment of plasma membrane active zones where vesicle docking and membrane fusion occur [7].

The Stx1 family contains two major neuronal isoforms, Stx1A and Stx1B. Stx1A has been extensively investigated in vitro, in vivo, and in silico and has served as a model system for understanding SNARE-mediated membrane fusion [8,9]. In contrast, Stx1B, although functionally redundant in part, has received substantially less mechanistic investigation despite its important physiological roles. Genetic deletion or knockdown of Stx1B causes reduced spontaneous neurotransmitter release, impaired fast evoked release, and decreased postnatal survival [10,11,12,13,14].

Despite extensive studies on the molecular mechanisms of membrane fusion, how Stx1 isoforms regulate upstream fusion processes such as membrane organization, docking, and SNARE assembly remains incompletely understood. In particular, Stx1 clustering has been observed in multiple experimental studies [7,15,16,17,18,19,20] as well as in an in silico study [21], where cluster formation occurs spontaneously and depends on local Stx concentration. In addition, Stx1A JMD was applied to induce formation of polyanionic phosphatidylinositol 4,5-bisphosphate (PIP_2_) domains recruiting Munc13 [22]. However, the functional significance of Stx1 clustering, potentially regulated by PIP_2_ [15,19,20,23], remains controversial. Some studies suggest that Stx1 clustering promotes vesicle docking and membrane fusion by organizing active membrane domains [16,19,20], whereas others propose that Stx1 clusters function as reservoir-like assemblies from which Stx molecules dissociate during SNARE complex assembly [15,23].

More recently, a hypothesis proposed by the Josep Rizo laboratory suggested that the JMD–TMD regions of Syb2 and Stx1 may facilitate membrane fusion through detergent-like membrane perturbation effects [24,25]. In this model, local membrane disorder induced by SNARE transmembrane regions promotes transient exposure of hydrophobic lipid tails to the surrounding environment, thereby facilitating hydrophobic contacts between opposing membranes and promoting stalk formation during fusion initiation. Consistent with this idea, previous studies have shown that hydrophobic mismatch between transmembrane domains and lipid bilayers can induce local membrane disorder [26], and computational studies further suggest that transmembrane-domain-induced membrane thinning lowers the energetic barrier for membrane fusion [27]. Together, these observations raise the possibility that local membrane disorder generated by SNARE transmembrane regions may contribute directly to membrane fusion initiation.

To investigate how Stx1 isoforms regulate membrane disorder and clustering behavior, and to examine how Stx1 TMD palmitoylation and PIP_2_ presence modulate these processes, we performed MARTINI cgMD simulations using systems containing 1, 2, 4, 6, 8, or 10 copies of the Stx1A or Stx1B JMD–TMD region embedded in a ~20 × 20 nm^2^ plasma membrane containing ~1500 lipids. To specifically investigate membrane organization mediated by the JMD–TMD region, the SNARE domain (SND) and Habc domain were excluded to reduce system complexity.

Our simulations revealed that Stx1 JMD–TMD regions induce local membrane lipid disorder primarily through hydrophobic mismatch, with Stx1A generating stronger and more spatially extended membrane perturbations than Stx1B. The magnitude of local lipid disorder remained largely independent of palmitoylation and PIP_2_ depletion. Stx1 proteins spontaneously formed oligomeric assemblies once sufficient protein density was reached, with clustering behavior regulated by both PIP_2_ presence and palmitoylation. Presence of PIP_2_ promoted clustering and palmitoylation suppressed higher-order oligomerization. Stx1B further exhibited a modestly greater propensity for oligomerization than Stx1A. Together, these findings support a unified framework in which hydrophobic mismatch, palmitoylation, and electrostatic lipid interactions cooperatively regulate membrane organization, Stx1 clustering dynamics, and transitions between reservoir-like assemblies and fusion-competent SNARE states during membrane fusion.

## 2. Results

### 2.1. Stx1 Induces Local Lipid Disorder Associated with Hydrophobic Mismatch

**Figure 1:**
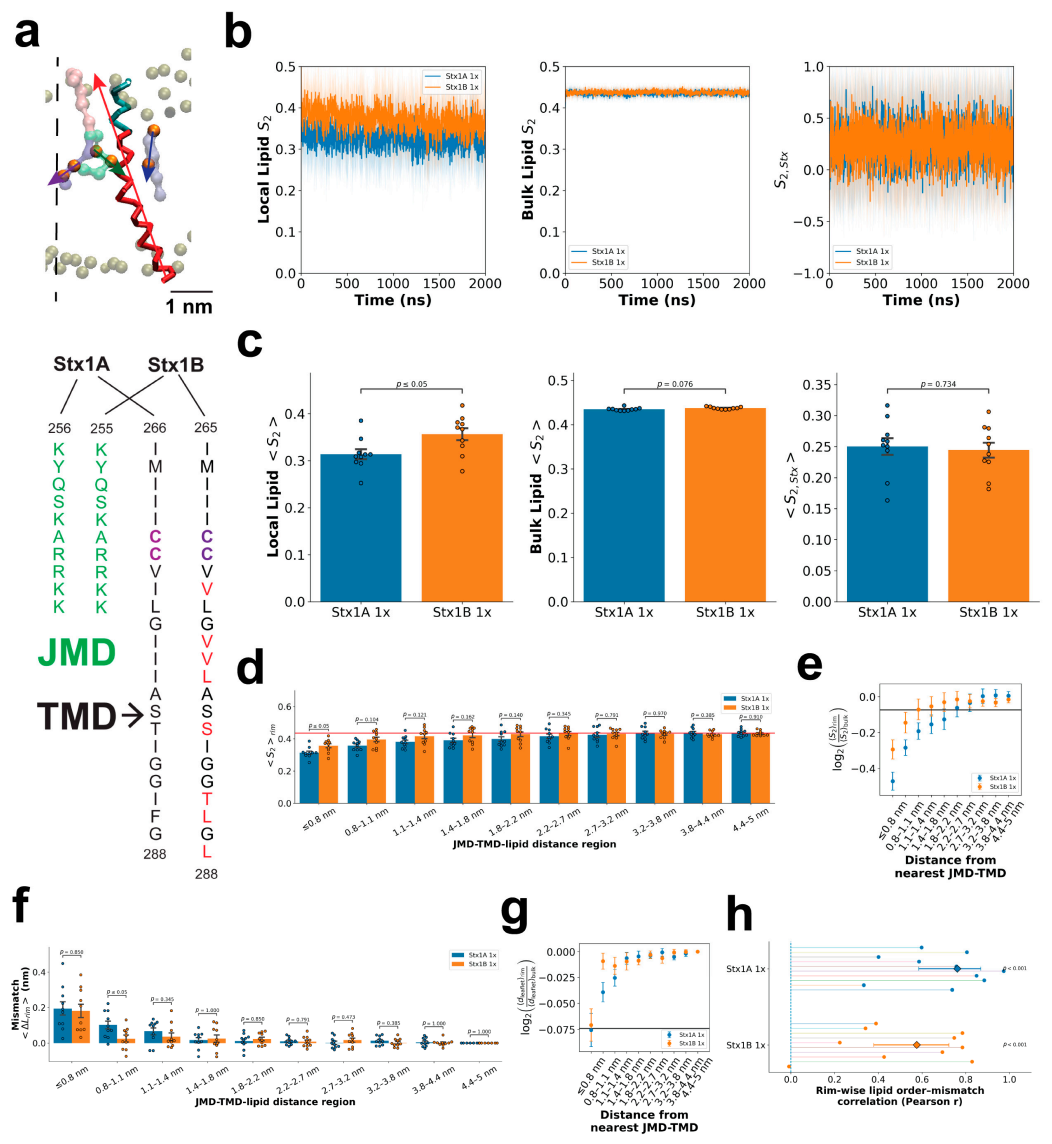
Stx1A induces stronger and broader local lipid disorder than Stx1B. (**a**) (Top) Representative simulation snapshot of a Stx1A JMD–TMD embedded in a plasma membrane at 1999 ns. Red: Stx1A TMD backbone beads; Cyan: Stx1A JMD backbone beads; brown: phospholipid PO_4_ beads; pink: representative head group beads of PIP_2_ interacting with the Stx1A JMD-TMD; purple and green: two lipid tails in the PIP_2_ molecule; blue: representative cholesterol interacting with Stx1A JMD-TMD. Orange: the second and the third beads of the two lipid tails in the PIP_2_ molecule and the ROH and R5 beads in the cholesterol molecule. Purple, green and blue arrows: vector connected by orange beads to calculate $\theta_{i}$ related to the membrane normal (dash line). Each lipid except cholesterol contributes two $\theta_{i}$ because of two lipid tails. For cholesterol, only one $\theta_{i}$ was calculated. Red arrow: vector of TMD to calculate TMD tilting angle. (Bottom) Sequence alignment of the Stx1A and Stx1B JMD–TMD regions, highlighting the amino acid differences within the TMDs. (**b**) Average trajectories of the local lipid order parameter for Stx-bound lipids (left), bulk membrane lipid order parameter calculated from all lipids in the simulation box (middle), and Stx1 TMD tilting order parameter (right) from *n* = 10 simulations of Stx1A (blue) and Stx1B (orange). Shaded regions indicate mean ± SD. (**c**) Averaged local lipid order parameters for local Stx-bound lipids (left), bulk membrane lipid order parameters (middle), and Stx1 TMD tilting order parameters (right) calculated from the final 100 ns of the simulations. Individual dots represent values from independent simulations. *p*-values were calculated using the Mann–Whitney U test. All statistics were listed in Table S1. (**d**) Radial analysis of lipid order parameters as a function of the distance from the Stx1 JMD–TMD region. The red horizontal line indicates the bulk membrane lipid order parameter. All statistics are listed in Table S2. (**e**) $\log_{2}\left(\frac{{\langle S_{2}\rangle }_{rim}}{{\langle S_{2}\rangle }_{bulk}}\right)$ values related to the bulk lipid ordering parameters at different radial distances with the bulk membrane lipid order parameter using the last 100 ns of all *n* = 10 simulations. Stx1B-induced lipid disorder became indistinguishable from bulk membrane ordering beyond ~1.4 nm from the Stx1 JMD–TMD region, whereas Stx1A-induced lipid disorder extended to ~2.7 nm, indicating an approximately twofold broader disordering range. Black line: Fold change of 95% of the bulk lipid ordering parameters. All statistics are listed in Table S3. Error bars: mean ± SEM. (**f**) Average local hydrophobic mismatch proxy, Δ*L*, within successive annular rims surrounding single-copy Stx1A and Stx1B JMD–TMD regions, calculated from the final 100 ns of the simulations. Δ*L* was estimated as the difference between the bulk membrane interleaflet headgroup distance (defined as the average distance within the 4.4–5.0 nm annular rim) and the local interleaflet headgroup distance measured within each annular rim. Larger positive values of Δ*L* therefore indicate greater local membrane thinning relative to the bulk membrane. Individual dots represent independent simulations. Error bars indicate mean ± SEM. *p* values were calculated using the Mann–Whitney U test. (**g**) ${log}_{2}\left(\frac{{\langle d_{leaflet}\rangle }_{rim}}{{\langle d_{leaflet}\rangle }_{bulk}}\right)$ of the local interleaflet headgroup distance relative to the bulk membrane as a function of radial distance from the Stx1 JMD–TMD region, where $d_{leaflet}$ denotes the interleaflet distance. Similar to the lipid-ordering analysis in panel (**e**), local mismatch progressively decreased toward bulk membrane values with increasing distance from the protein. Black line: Fold change of 95% of the bulk local interleaflet headgroup distance. (**h**) Rim-wise Pearson correlation between the local lipid order parameter (panel (**d**)) and the local interleaflet headgroup distance calculated independently for all rims of each simulation using the final 100 ns. Blue symbols represent Stx1A 1× and orange symbols represent Stx1B 1×. Each circle represents one independent simulation, while diamonds indicate the Fisher *z*-transformed mean correlation with the corresponding 95% confidence interval. These analyses demonstrate a consistent positive correlation between local lipid ordering and interleaflet headgroup distance, indicating that stronger local lipid disorder is accompanied by increased local membrane thinning around the Stx1 JMD–TMD region.

To investigate whether the Stx1 JMD–TMD region induces local lipid disorder relative to distal bulk membrane lipids, we constructed simulation systems containing a single Stx1A or Stx1B JMD–TMD embedded in a ~20 × 20 nm^2^ plasma membrane containing ~1500 lipids (Figure 1a). For each system, *n* = 10 independent simulations of 2 μs duration were performed to ensure robust statistical sampling. Additional details regarding system construction and simulation protocols are provided in Section 4.

To quantify lipid ordering, we used the following lipid order parameter:

(1) $$S_{2}\left(t,\;r_{crit}\right)=\frac{1}{2}\langle 3\cos^{2}\theta_{i}-1\rangle ,$$

where 〈 〉 denotes averaging over all selected lipids, and $\theta_{i}$ is the angle between the lipid hydrophobic vectors formed by the second and third lipid beads for phospholipids (marked by the purple arrow and green arrow for the two hydrophobic tails, respectively, in Figure 1a, left) and the membrane normal (z-axis) for every selected lipid [21]. For cholesterol, a similar definition was used but the lipid hydrophobic vector was defined by connecting the R5 and ROH beads for cholesterols (the blue arrow in Figure 1a).

Stx1-bound lipids were defined using a cutoff distance of $r_{crit}=0.8\;nm$ at time t, whereas all lipids in the membrane were included to calculate the bulk lipid order parameter. Similarly, the Stx1 TMD tilting order parameter was calculated as

(2) $$S_{2,\;Stx}\left(t\right)=\frac{1}{2}\langle 3\cos^{2}\theta_{TMD,i}\left(t\right)-1\rangle ,$$

where $\theta_{TMD,i}\left(t\right)$ is the tilt angle between the vector fit to all of the backbone beads of the TMD residues of the *i*th Stx1 JMD–TMD fragment and the membrane normal at time *t*, and 〈 〉 denotes averaging over all Stx1 molecules in the simulation system.

Higher *S*_2_ values indicate more ordered and tightly packed lipid or TMD conformations, whereas lower *S*_2_ values indicate increased tilting away from the membrane normal and greater exposure of hydrophobic regions to the surrounding environment.

The averaged trajectories from the *n* = 10 simulations showed no systematic temporal drift in either local or global lipid ordering parameters or in the Stx1 TMD tilting order parameter (Figure 1b), indicating that the systems were well equilibrated preceding the production simulations.

Analysis of the final averaged lipid ordering parameters (Figure 1c, Table S1) revealed that lipids bound to Stx1 were substantially more disordered than bulk membrane lipids (0.314 ± 0.011 vs. 0.435 ± 0.001 for Stx1A and 0.357 ± 0.013 vs. 0.437 ± 0.001 for Stx1B; both *p* < 0.0002 from paired *t*-test). In contrast, no significant differences were observed between the bulk lipid order parameters and the Stx1A/Stx1B TMD tilting order parameters, indicating that Stx1A and Stx1B primarily induce localized lipid disorder without substantially altering global membrane ordering.

Importantly, lipids surrounding Stx1A were significantly more disordered than those surrounding Stx1B, with a ~1.137 ± 0.057-fold reduction in the local lipid order parameter (*p* < 0.05, Mann–Whitney U test). Then we estimated the tilting order parameters and found that Stx1A and Stx1B exhibited similar TMD tilting order parameters. So the stronger lipid disordering induced by Stx1A is unlikely to result from differences in TMD tilting dynamics. For further details, please see Table S1.

Consistent with this interpretation, radial analyses of lipid ordering revealed that lipid order parameters approached bulk membrane values (less than 5% related decrease) only at distances greater than ~2.7 nm from the Stx1A JMD–TMD, whereas this recovery occurred at ~1.4 nm for Stx1B (Figure 1d). For more information, please see Table S2. These observations were further supported by the radial profiles of the $\log_{2}\left(\frac{{\langle S_{2}\rangle }_{rim}}{{\langle S_{2}\rangle }_{bulk}}\right)$ in local lipid order parameters relative to the bulk membrane (Figure 1e), which consistently demonstrated a longer spatial range of membrane perturbation surrounding Stx1A than Stx1B.

To further examine whether the observed lipid disorder is associated with local membrane hydrophobic mismatch, we quantified the local mismatch parameter, Δ*L*, defined as the difference between the bulk membrane interleaflet distance and the local interleaflet distance within each annular rim (Figure 1f). Stx1A exhibited a modest tendency toward greater local mismatch than Stx1B within the membrane regions immediately surrounding the protein, whereas the difference gradually diminished with increasing radial distance from the Stx1 JMD–TMD region. Similar to the lipid-ordering analysis, the relative interleaflet distance progressively recovered toward the bulk membrane value with increasing distance from the protein (Figure 1g), indicating that membrane thinning induced by the Stx1 JMD–TMD region is spatially localized. Finally, rim-wise Pearson correlation analyses performed independently for each simulation demonstrated a consistent positive correlation between local lipid order parameters and local interleaflet distances for both Stx1A and Stx1B (Figure 1h). Together, these observations indicate that local membrane thinning and lipid disorder exhibit closely associated spatial profiles, supporting the interpretation that the stronger membrane perturbation induced by Stx1A is associated with a larger local hydrophobic mismatch.

### 2.2. Intrinsic TMD Sequence Establishes Local Lipid Disorder, Whereas Membrane Perturbations Modulate Its Spatial Extent

**Figure 2:**
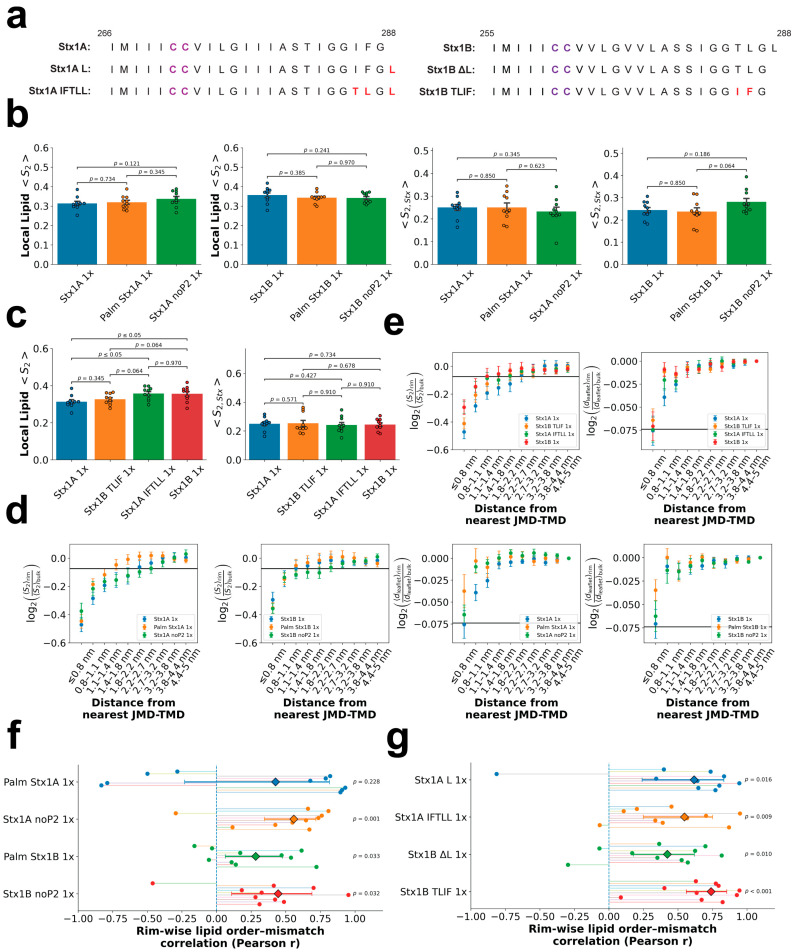
Palmitoylation, PIP_2_ depletion, and C-terminal sequence modifications differentially regulate the magnitude and spatial extent of Stx1-induced membrane perturbation. (**a**) Sequence alignments of the C-terminal Stx1A and Stx1B TMD mutants used to investigate the sequence determinants of Stx1-induced lipid disorder. (**b**) Average local lipid order parameters of Stx-associated lipids and average Stx1 TMD tilting order parameters calculated over the final 100 ns of the simulations. Neither palmitoylation nor PIP_2_ depletion substantially altered the overall magnitude of local lipid disorder or the Stx1 TMD tilting order parameter for either isoform. (**c**) Average local lipid order parameters and Stx1 TMD tilting order parameters for the C-terminal sequence mutants. Addition or deletion of the terminal leucine produced only modest effects (Figure S1). In contrast, the Stx1A IFTLL mutant exhibited a small but statistically significant increase in the local lipid order parameter relative to wild-type Stx1A, corresponding to reduced lipid disorder and a shift toward the wild-type Stx1B phenotype. The reciprocal Stx1B TLIF substitution produced only a partial shift toward Stx1A. Individual dots in (**b**,**c**) represent independent simulations. Error bars indicate mean ± SEM. *p* values were calculated using the Mann–Whitney U test. All statistical comparisons are summarized in Table S1. (**d**) Radial profiles of relative lipid ordering and interleaflet distance surrounding wild-type, palmitoylated, and PIP_2_-depleted Stx1A and Stx1B JMD–TMD regions. Palmitoylation reduced the spatial extent of lipid disorder surrounding Stx1A, whereas PIP_2_ depletion was associated with a modest broadening of the disorder profile surrounding Stx1B. The corresponding interleaflet-distance profiles showed similar spatially localized membrane perturbations. Black line: Fold change of 95% of the bulk lipid ordering parameters or bulk interleaflet distances. (**e**) Radial profiles of relative lipid ordering and interleaflet distance for the C-terminal sequence mutants. Addition or deletion of the terminal leucine produced partial shifts toward the opposite isoform. Replacement of the Stx1A C-terminal sequence with IFTLL nearly reproduced the Stx1B radial disorder profile, whereas the reciprocal Stx1B TLIF substitution produced only a partial shift toward Stx1A. Black line: Fold change of 95% of the bulk lipid ordering parameters or bulk interleaflet distances. (**f**) Rim-wise Pearson correlations between the local lipid order parameter and interleaflet distance for the palmitoylation and PIP_2_-depletion systems. (**g**) Rim-wise Pearson correlations between the local lipid order parameter and interleaflet distance for the C-terminal sequence mutants. Each circle in (**f**,**g**) represents the correlation coefficient calculated from one independent simulation. Colors correspond to the different simulation systems listed on the y-axis. Diamonds indicate the Fisher *z*-transformed mean correlation with the corresponding 95% confidence interval, and the blue dashed vertical line denotes zero correlation. Positive associations were generally preserved across the mutant systems, supporting a close spatial relationship between local membrane thinning and lipid disorder.

Stx1 palmitoylation has been implicated in spontaneous neurotransmitter release [28] whereas PIP_2_ interactions are known to regulate Ca^2+^-dependent membrane fusion [29]. To evaluate the robustness of Stx1-induced membrane disorder, we compared two perturbations that potentially influence membrane interactions: TMD palmitoylation, and PIP_2_ depletion. Then, to further evaluate whether specific C-terminal sequence motifs contribute to the distinct membrane-disordering properties of Stx1A and Stx1B, we examined four C-terminal sequence substitutions, Stx1A L, Stx1A IFTLL, Stx1B Δ*L* and Stx1B TLIF (Figure 2a). We quantified both the local lipid order parameters and the Stx1 TMD tilting order parameters in single-copy Stx1A and Stx1B systems together with their radial spatial profiles.

None of these perturbations substantially altered either the overall magnitude of local lipid disorder or the average Stx1 TMD tilting order parameter (Figure 2b). Addition or deletion of the terminal leucine produced only modest changes in the local lipid order parameter (Figure S1). In contrast, replacing the C-terminal TMD sequence of Stx1A with the corresponding Stx1B sequence (Stx1A IFTLL) resulted in a small but statistically significant increase in the lipid order parameter, shifting the membrane phenotype toward that of wild-type Stx1B (Figure 2c). The reciprocal Stx1B TLIF substitution produced only a partial shift toward the Stx1A phenotype. The average Stx1 TMD tilting order parameter was not affected by any of these mutations.

We next examined whether these perturbations altered the spatial extent of membrane disorder. Radial analyses of the relative lipid order parameter showed that TMD palmitoylation markedly reduced the spatial extent of membrane disorder surrounding Stx1A, whereas PIP_2_ depletion produced only minimal changes (Figure 2d, left). In contrast, palmitoylation exerted little effect on Stx1B, whereas PIP_2_ depletion exhibited a modest tendency to broaden the radial disorder profile. Similar trends were observed for the corresponding radial interleaflet-distance profiles, indicating that membrane thinning changed in parallel with the radial lipid-disorder profiles (Figure 2d, right).

Among the C-terminal mutants, insertion of a terminal leucine into Stx1A produced only modest changes in the radial disorder profile (Figure S1b). However, replacing the C-terminal sequence of Stx1A with the corresponding Stx1B sequence (Stx1A IFTLL) shifted the radial disorder profile close to that of wild-type Stx1B (Figure 2e, left). Conversely, deletion of the terminal leucine from Stx1B caused only minor changes, whereas the reciprocal TLIF substitution produced a partial shift toward the Stx1A radial disorder profile (Figure 2e, left). Together, these observations indicate that the C-terminal TMD sequence plays a major role in determining the isoform-specific spatial extent of membrane disorder, whereas the terminal leucine alone is insufficient to account for the full phenotypic difference. Similar trends were observed for the radial interleaflet-distance profiles, indicating that changes in membrane thinning closely paralleled the corresponding changes in radial lipid disorder (Figure 2e, right).

Finally, rim-wise Pearson correlation analyses performed independently for each simulation consistently revealed positive correlations between local lipid order parameters and local interleaflet distances under nearly all conditions (Figure 2f,g). These observations further support a close spatial association between local membrane thinning and lipid disorder and are consistent with the interpretation that hydrophobic mismatch contributes to the membrane perturbations induced by the Stx1 JMD–TMD region.

Collectively, these results demonstrate that the overall magnitude of Stx1-induced lipid disorder is largely determined by the intrinsic TMD sequence. Palmitoylation and PIP_2_ depletion exert comparatively modest effects on local lipid disorder but selectively modulate the spatial extent of membrane perturbation in an isoform-dependent manner. C-terminal sequence substitutions partially shifted the membrane phenotype toward the opposite isoform, with Stx1A IFTLL producing a Stx1B-like level of local lipid disorder, whereas the reciprocal TLIF substitution was insufficient to fully reproduce the Stx1A phenotype. Finally, the consistently positive correlations between local lipid disorder and hydrophobic mismatch, together with the reciprocal sequence substitutions, support a model in which local hydrophobic mismatch is closely associated with Stx1-induced membrane perturbation, while the intrinsic TMD sequence fine-tunes the magnitude and spatial extent of lipid disorder.

### 2.3. Multiple Copies of Stx1 JMD–TMD Spontaneously Form Clusters

Next, we investigated whether multiple copies of the Stx1 JMD–TMD region spontaneously cluster within the plasma membrane, potentially generating local Stx reservoirs prior to SNARE complex assembly and membrane docking.

**Figure 3:**
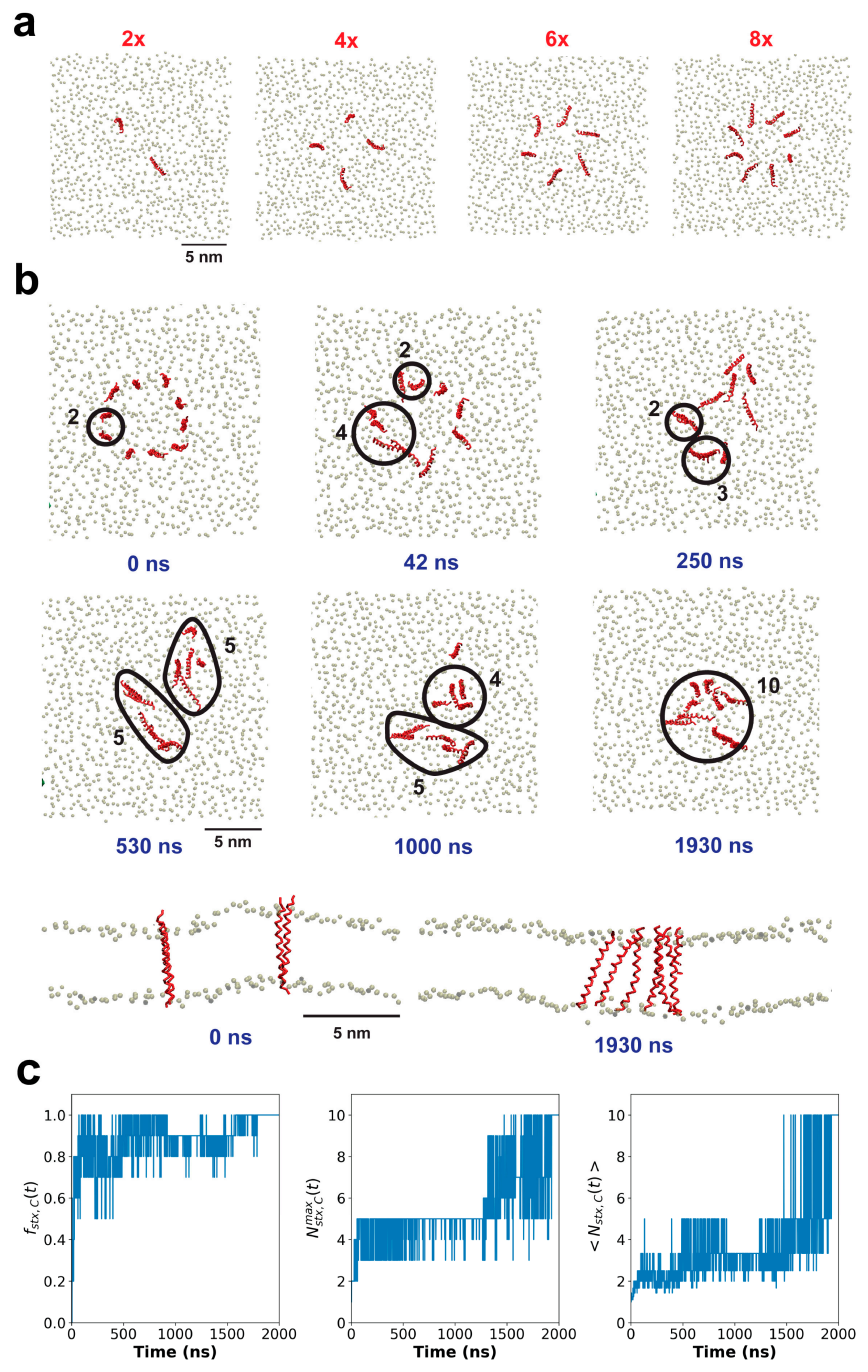
Stx1 JMD–TMD fragments spontaneously assemble into dynamic oligomeric clusters. (**a**) Initial configurations of systems containing 2, 4, 6, and 8 copies of Stx1A JMD–TMD embedded in ~20 × 20 nm^2^ plasma membrane models. Proteins were initially distributed within a circular region (~6 nm diameter) to facilitate observation of clustering dynamics while avoiding immediate formation of large oligomers. Red: Stx1A JMD–TMD; brown: lipid phosphate (PO_4_) beads. (**b**) Representative clustering trajectory from a simulation containing 10 copies of Stx1A JMD–TMD. Pairs of Stx1 JMD–TMDs were considered connected when the minimum backbone pairwise distance was ≤1.0 nm. Clusters identified from these pairwise contacts are encircled, and numbers indicate cluster size. Representative side-view snapshots of the same trajectory at 0 ns and 1930 ns are shown at the bottom to illustrate the orientations of Stx1 JMD–TMDs relative to the membrane. Additional clustering dynamics are shown in Video S1. (**c**) Time courses of the fraction of Stx1 JMD–TMD molecules participating in clusters $f_{stx,\;C}\left(t\right)$, the maximum cluster size $N_{stx,C}^{max}(t)$, and the mean cluster size for the simulation $\langle N_{stx,C}(t)\rangle$ shown in (**b**). Scale bars: 5 nm.

To examine clustering behavior at different protein concentrations, systems containing 2, 4, 6, 8, or 10 copies of Stx1 JMD–TMD were embedded in ~20 × 20 nm^2^ plasma membranes (Figure 3a). Representative clustering dynamics from a simulation containing 10 copies of Stx1A are shown in Figure 3b. The corresponding side-view snapshots at the beginning (0 ns) and the end (1930 ns) of the same trajectory shown at the bottom of Figure 3b illustrate the orientations of clustered Stx1 JMD–TMD molecules relative to the membrane. Clusters were defined as connected groups of Stx1 JMD–TMD molecules whose minimum backbone pairwise distances were ≤1.0 nm.

Small oligomers formed rapidly after the start of the simulations (Figure 3b). Dimers and trimers appeared within tens of nanoseconds and subsequently associated into larger oligomeric assemblies. However, cluster formation was highly dynamic, with repeated association and dissociation events occurring throughout the trajectory. Intermediate-sized oligomers frequently merged, separated, and reorganized before eventually forming larger assemblies. After prolonged sampling, oligomers containing all 10 Stx1A molecules were occasionally observed and ultimately became stabilized near the end of the simulation.

These observations suggest that Stx1 JMD–TMD clustering is reversible and hierarchical, proceeding from monomers to dimers and trimers, followed by assembly into intermediate-sized oligomers and, under sufficiently high protein density, larger cluster states, consistent with ref. [7].

This behavior is consistent with the time courses of the fraction of Stx1 JMD–TMD molecules participating in clusters, $f_{stx,\;C}(t)$; the maximum cluster size, $N_{stx,C}^{max}(t)$; and the mean cluster size, $\langle N_{stx,C}\left(t\right)\rangle$ (Figure 3c). The fraction of Stx1 molecules participating in clusters increased rapidly and reached high values within approximately 100 ns (Figure 3c, left), indicating rapid formation of low-order oligomers. In contrast, larger oligomers formed more slowly and exhibited substantial fluctuations over time, as reflected by the intermittent trajectories of the maximum and mean cluster sizes (Figure 3c, middle and right). These fluctuations indicate continual association and dissociation of higher-order oligomers. Large oligomeric assemblies became increasingly visible after approximately 1.5 μs, suggesting that formation of extensive Stx1 clusters occurs through progressive hierarchical assembly rather than direct aggregation of isolated monomers.

### 2.4. Stx1 JMD–TMD Clustering Increases with Protein Concentration

**Figure 4:**
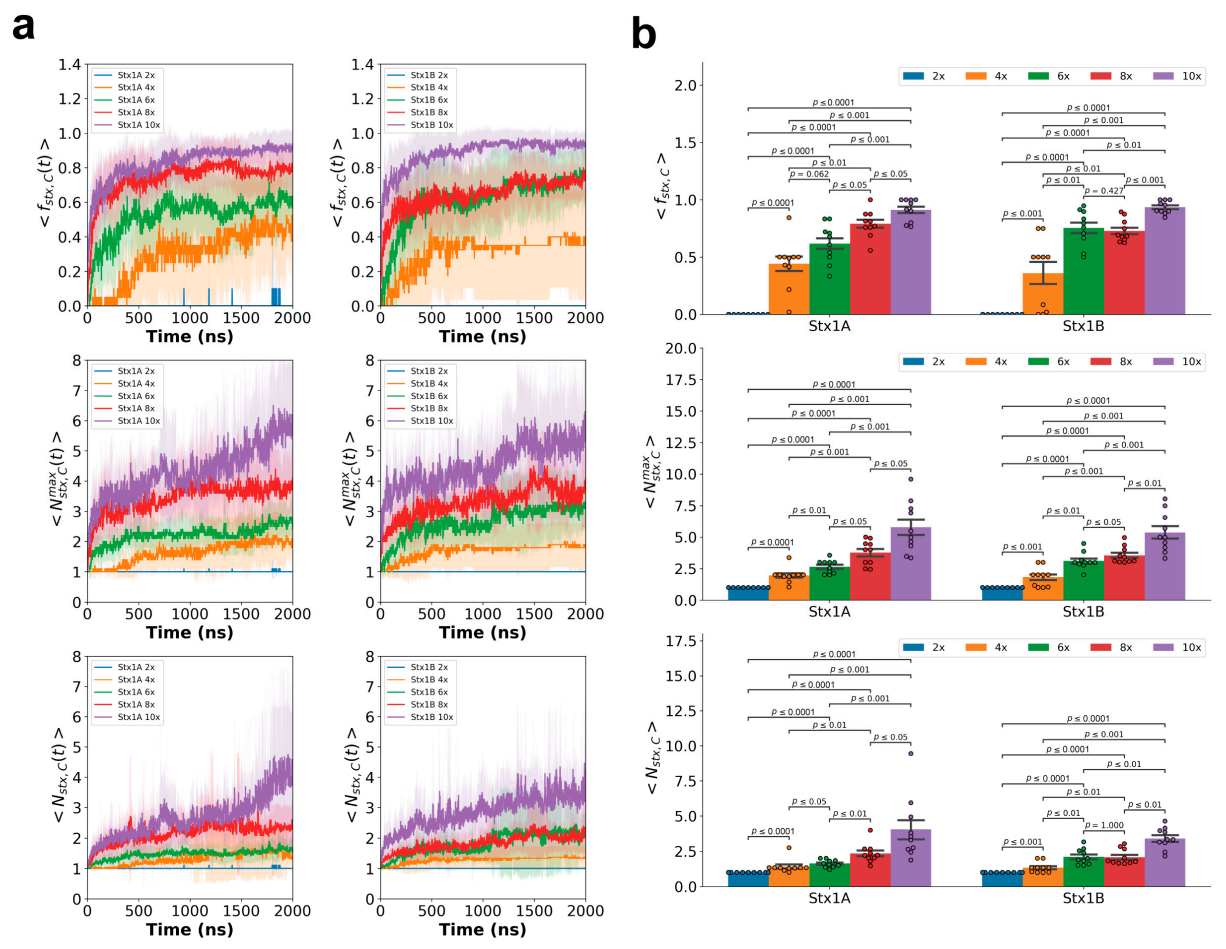
Stx1 JMD–TMD clustering increases with protein density. (**a**) Average trajectories from *n* = 10 simulations showing the fraction of Stx1 JMD–TMD molecules participating in clusters $\langle f_{stx,C}\left(t\right)\rangle$, the maximum cluster size $\langle N_{stx,C}^{max}(t)\rangle$, and the mean cluster size $\langle N_{stx,C}(t)\rangle$ for systems containing 2, 4, 6, 8, and 10 copies of Stx1A or Stx1B. Higher-order oligomerization emerged when at least four copies of Stx1 JMD–TMD were present in the membrane. Cluster growth proceeded progressively through hierarchical assembly of smaller oligomers. Shaded regions indicate mean ± SD. (**b**) Final values of the fraction of Stx1 molecules participating in clusters, maximum cluster size, and mean cluster size calculated from the last 100 ns of *n* = 10 simulations. Individual circles represent independent simulations. Error bars: mean ± SEM. *p* values were calculated using the Mann–Whitney U test. All statistics are provided in Table S4. Color codes are for different number of copies of Stx JMD-TMD fragments.

We next examined how Stx1 JMD–TMD clustering depends on protein concentration. Average trajectories of the fraction of Stx1 molecules participating in clusters $\langle f_{stx,C}\left(t\right)\rangle$, the maximum cluster size $\langle N_{stx,C}^{max}(t)\rangle$, and the mean cluster size $\langle N_{stx,C}(t)\rangle$ revealed progressive hierarchical assembly for both Stx1A and Stx1B (Figure 4a).

Stable dimers were rarely observed in systems containing only two copies of Stx1 JMD–TMD. In contrast, systems containing at least four copies rapidly formed dimers and trimers within the first few hundred nanoseconds, followed by slower growth into larger oligomeric assemblies. Consistent with the representative trajectory shown in Figure 3, cluster growth exhibited two distinct phases: a rapid initial assembly phase dominated by low-order oligomers and a slower maturation phase characterized by association and reorganization of pre-existing clusters. Increasing Stx1 copy number accelerated cluster formation and promoted the emergence of larger oligomeric assemblies.

Quantitative analysis of the final 100 ns of the simulations demonstrated a strong protein concentration dependence of clustering (Figure 4b). For Stx1A, the fraction of molecules participating in clusters increased progressively with copy number, reaching 91.3% ± 3.1% at 10 copies of Stx1A. Similar behavior was observed for Stx1B (93.5% ± 1.8%). The maximum cluster size and mean cluster size also increased with increasing protein concentration for both isoforms, indicating progressive assembly of larger oligomeric states at higher membrane densities. There were no significant differences in oligomerization between Stx1A and Stx1B (Figure S2).

Together, these results indicate that Stx1 JMD–TMD oligomerization is strongly dependent on protein concentration and proceeds through progressive hierarchical assembly of increasingly larger oligomeric states. Protein concentration appears to be the primary determinant of clustering behavior, whereas isoform-specific differences are comparatively modest, if any.

### 2.5. Palmitoylation and PIP_2_ Depletion Suppress Higher-Order Oligomerization More Strongly in Stx1A than in Stx1B

**Figure 5:**
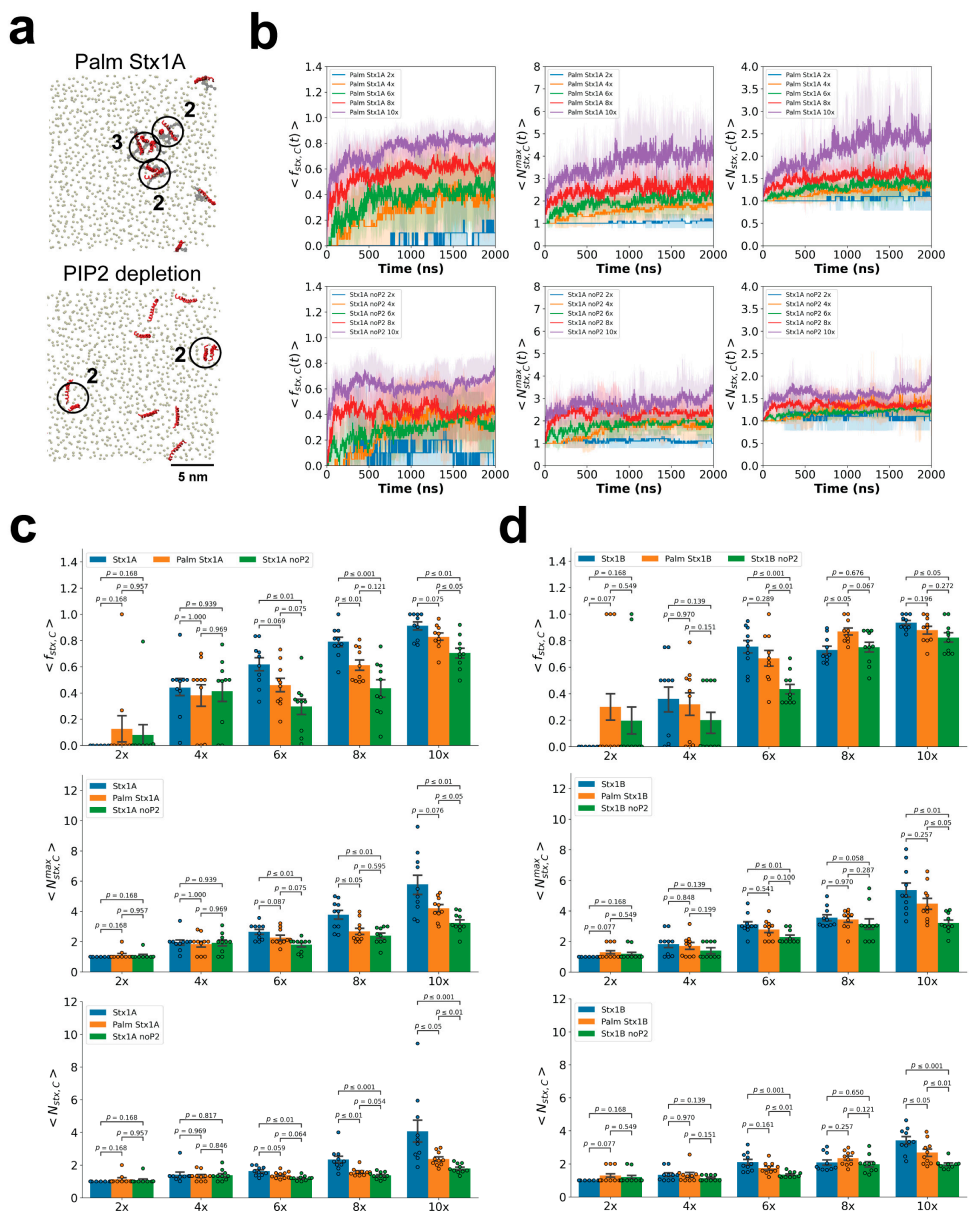
Palmitoylation and PIP_2_ depletion limit formation of higher-order Stx1 clusters. (**a**) Representative final clustering states of simulations containing 10 copies of palmitoylated Stx1A (Palm Stx1A) or Stx1A in PIP_2_-depleted membranes (Stx1A noP2). Clusters identified from these pairwise contacts are encircled, and numbers indicate cluster size. Red: Stx1 JMD–TMD; brown: lipid phosphate (PO_4_) beads; gray: palmitoyl chains. Scale bar: 5 nm. For more visualization, please see Videos S2 and S3. (**b**) Average trajectories from *n* = 10 simulations showing the fraction of Stx1 molecules participating in clusters $\langle f_{stx,C}\left(t\right)\rangle$, the maximum cluster size $\langle N_{stx,C}^{max}(t)\rangle$, and the mean cluster size $\langle N_{stx,C}(t)\rangle$, for systems containing 2, 4, 6, 8, and 10 copies of Palm Stx1A or Stx1A in PIP_2_-depleted membranes. Shaded regions indicate mean ± SD. Color codes are for different number of copies of Stx JMD-TMD fragments. (**c**,**d**) Final values of the fraction of Stx1 molecules participating in clusters, maximum cluster size, and mean cluster size calculated from the final 100 ns of *n* = 10 simulations for Stx1A (**c**) and Stx1B (**d**). Individual circles represent independent simulations. Error bars indicate mean ± SEM. *p* values were calculated using the Mann–Whitney U test. All statistics are provided in Table S4. Blue: WT Stx1; orange: palmitoylated Stx1; green: Stx1 in PIP_2_ depleted membrane.

Next, we investigated how TMD palmitoylation and membrane PIP_2_ regulate Stx1 JMD–TMD clustering. Representative simulations containing 10 copies of Stx1A revealed that both palmitoylation and PIP_2_ depletion suppressed formation of large oligomeric assemblies (Figure 5a, Videos S2 and S3). In the palmitoylated system, a trimer and two dimers were observed near the end of the simulation, whereas only two dimers remained in the PIP_2_-depleted membrane. These observations suggest that both perturbations inhibit higher-order oligomerization, with PIP_2_ depletion producing the stronger effect.

This behavior was consistent with the average clustering trajectories (Figure 5b and Figure S3a). In palmitoylated Stx1A systems, rapid formation of low-order oligomers was preserved, but subsequent growth into larger assemblies was attenuated. In contrast, PIP_2_ depletion largely abolished the slow-growth phase associated with maturation of higher-order oligomers, indicating a stronger suppression of cluster expansion.

Quantitative analysis of the final 100 ns of the simulations confirmed these observations (Figure 5c). Palmitoylation significantly reduced the mean cluster size at 8 and 10 copies of Stx1A and decreased both clustering fraction and maximum cluster size at 8 copies. However, little effect was observed at ≤6 copies, suggesting that palmitoylation primarily suppresses assembly of higher-order oligomers rather than formation of dimers and trimers.

PIP_2_ depletion produced a substantially stronger phenotype. Significant reductions in clustering fraction, maximum cluster size, and mean cluster size were observed once systems contained at least six copies of Stx1A. At 10 copies, the maximum cluster size was reduced to approximately three molecules, indicating that formation of oligomers larger than trimers was strongly inhibited in the absence of PIP_2_.

Protein-concentration-dependent analysis further revealed that the suppressive effects of both palmitoylation and PIP_2_ depletion could be partially overcome at high Stx1A concentrations (Figure S3b), indicating that increased local protein density promotes clustering even under conditions unfavorable for oligomerization.

In contrast, Stx1B exhibited substantially greater resistance to both perturbations (Figure 5d and Figure S3c). Palmitoylation produced little reduction in clustering fraction or maximum cluster size and only decreased mean cluster size at 10 copies. PIP_2_ depletion significantly reduced clustering metrics only at selected protein concentrations, while clustering behavior at 8 copies remained largely comparable to that of wild-type Stx1B. Notably, clustering properties of Stx1B approached saturation at 8 copies, whereas Stx1A continued to increase between 8 and 10 copies.

Together, these results indicate that both palmitoylation and PIP_2_ depletion suppress formation of higher-order Stx1 oligomers, but these effects are substantially stronger for Stx1A than for Stx1B. The enhanced resistance of Stx1B to oligomerization suppression may reflect intrinsic differences in the C-terminal region of the transmembrane domain and suggests isoform-specific regulation of membrane organization.

### 2.6. Stx1 JMD–TMD Regions Actively Recruit PIP_2_ and Promote Local PIP_2_ Enrichment During Clustering

**Figure 6:**
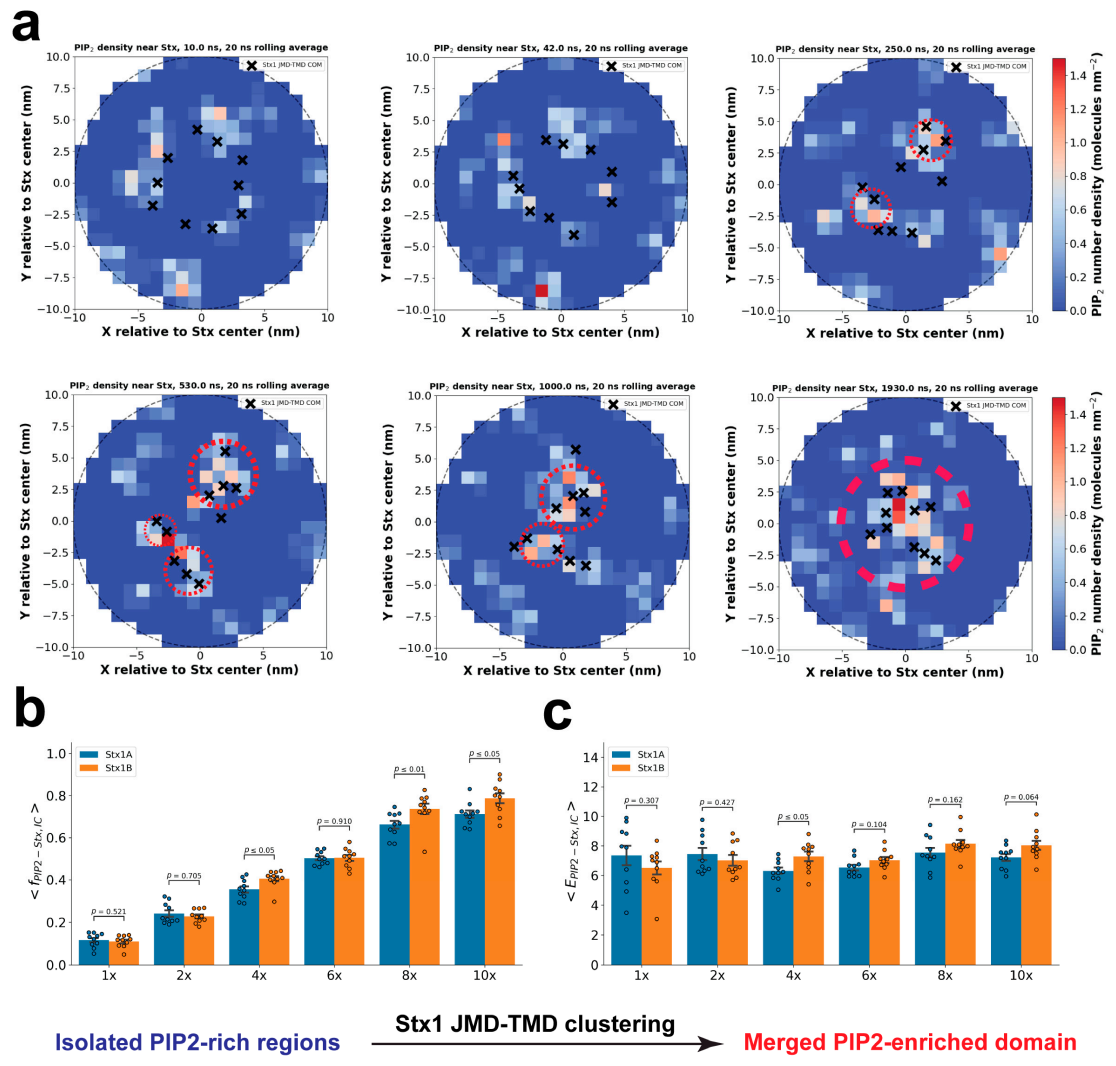
PIP_2_ is progressively recruited into locally disordered membrane domains generated by Stx1 JMD–TMD clustering. (**a**) Representative PIP_2_ density heatmaps from a simulation containing ten copies of the Stx1A JMD–TMD region. The snapshots correspond to the frames shown in Figure 3b. Black crosses indicate the centers of mass of individual Stx1A JMD–TMD regions. Red dashed circles highlight representative PIP_2_-enriched regions. As Stx1A molecules progressively assemble into oligomeric clusters, they recruit PIP_2_ molecules and promote the merging of initially isolated PIP_2_-rich regions into a larger PIP_2_-enriched membrane domain, resulting in progressive PIP_2_ redistribution toward the cluster center. All heatmaps are displayed using the same color scale. PIP_2_ density (molecules nm^−2^) was calculated using a 20 ns rolling average (±10 ns around each frame). Only PIP_2_ molecules located within 10 nm of the center of mass of the ten Stx1A JMD–TMD molecules were included in the analysis. For more details, please see Video S1. (**b**) Fraction of intracellular (IC) leaflet PIP_2_ molecules bound by at least one Stx1A or Stx1B JMD region in the corresponding systems, averaged over the final 100 ns of each simulation. PIP_2_ molecules were considered associated with Stx when any PIP_2_ bead was within 0.8 nm of the JMD region. All statistics are provided in Table S5. (**c**) Local PIP_2_ enrichment surrounding Stx1A or Stx1B JMD regions, calculated as the ratio between the local PIP_2_ fraction within the JMD binding region and the average PIP_2_ fraction of the IC leaflet. Values in (**b**,**c**) were averaged over the final 100 ns of each simulation. Individual circles represent independent simulations. *p* values were calculated using the Mann–Whitney U test. All statistics are provided in Table S5. Blue bars: Stx1A, orange bars: Stx1B.

Since PIP_2_ depletion strongly inhibited higher-order Stx1 JMD–TMD oligomerization (Figure 5), we next investigated how PIP_2_ is reorganized during the clustering process. Analysis of the spatial distribution of PIP_2_ density revealed that the localized PIP_2_-rich regions generated around individual Stx1A JMD–TMD molecules progressively coalesced as oligomerization proceeded (Figure 6a, Video S1). As neighboring protein clusters merged into larger assemblies, their associated PIP_2_-enriched regions also increasingly overlapped, ultimately forming a single confined PIP_2_-enriched membrane domain associated with the protein cluster.

We next quantified the fraction of intracellular (IC) leaflet PIP_2_ molecules bound by at least one Stx1 JMD region containing multiple basic residues (Figure 1a). Since PIP_2_ binding is primarily mediated by the polybasic JMD rather than the hydrophobic transmembrane domain, all subsequent analyses quantify PIP_2_ molecules associated with the JMD region. The average trajectories showed that PIP_2_ recruitment progressively increased during the simulations for higher Stx1 copy numbers (Figure S4a), consistent with the spatial redistribution observed in Figure 6a. As the number of Stx1 JMD–TMD copies increased, the bound PIP_2_ fraction increased from approximately 10% to ~70% for Stx1A and ~80% for Stx1B (Figure 6b), indicating progressive redistribution of PIP_2_ from the bulk membrane into Stx1-associated membrane regions. Under non-palmitoylated conditions, Stx1B exhibited a moderately greater propensity than Stx1A to recruit PIP_2_ at 4, 8, and 10 copies. Palmitoylation did not qualitatively alter this trend but produced modest isoform- and protein-copy-number-dependent effects on PIP_2_ recruitment (Figure S4b, Video S2).

To quantify the ability of the polybasic JMD to concentrate PIP_2_, we defined the PIP_2_ enrichment factor as

(3) $$E_{PIP2-Stx,IC}=\frac{f_{PIP2-Stx,\;\;IC}}{f_{PIP2,\;\;IC}},\;$$

where $f_{PIP2-Stx,IC}$ is the fraction of PIP_2_ molecules among all lipids bound to any Stx1 JMD within the IC leaflet, and $f_{PIP2,\;IC}$ is the corresponding bulk PIP_2_ fraction in the intracellular leaflet. Both non-palmitoylated Stx1A and Stx1B exhibited similar enrichment capacities, with $E_{PIP2-Stx,IC}$ remaining approximately 7 across all protein copy numbers (Figure 6c), indicating that the local PIP_2_ concentration associated with Stx1 JMD regions was approximately seven-fold higher than the average concentration in the IC leaflet. Palmitoylation produced only modest changes in PIP_2_ enrichment and these effects became more apparent only at higher protein copy numbers (Figure S4c).

Together, these results demonstrate that Stx1 clustering progressively redistributes PIP_2_ into Stx-associated membrane regions while maintaining a highly PIP_2_-enriched local membrane environment. These observations provide a mechanistic explanation for the strong dependence of higher-order Stx1 clustering on membrane PIP_2_ observed in Figure 5 by demonstrating that Stx clustering actively redistributes and concentrates PIP_2_ within Stx-associated membrane domains.

### 2.7. Multiple Copies of Stx1 Do Not Substantially Alter Local Lipid Disorder Induced by Individual JMD–TMD Regions

Finally, we investigated whether increasing Stx1 JMD–TMD copy number alters the magnitude of local membrane disorder generated by individual proteins. Because Stx1 fragments spontaneously formed oligomeric assemblies at higher protein densities, we asked whether clustering itself modifies local membrane organization or TMD dynamics.

To address this question, we repeated the analyses of local lipid order parameters and Stx1 TMD tilting order parameters for systems containing 1–10 copies of Stx1A or Stx1B (Figure S5a; Table S1). Despite pronounced differences in clustering behavior across protein concentrations (Figure 3, Figure 4 and Figure 5), both the local lipid order parameter and the Stx1 TMD tilting order parameter remained largely unchanged with increasing Stx1 copy number. Although several pairwise comparisons reached statistical significance, the corresponding changes in local lipid disorder were very small (generally <10%). Consistent with this observation, Kruskal–Wallis tests detected no significant overall effect of copy number on local lipid disorder for either Stx1A or Stx1B (*p* = 0.196 and *p* = 0.220, respectively). Likewise, no overall copy number dependence was observed for Stx1 TMD tilting disorder (Figure S5a).

A similar pattern was observed in palmitoylated and PIP_2_-depleted systems (Figure S5b,c). In PIP_2_-depleted Stx1A membranes, modest fluctuations in local lipid order parameters (~10%) were observed across copy numbers, whereas TMD tilting remained unchanged. However, these variations were substantially smaller than the differences observed between Stx1 isoforms and did not exhibit a monotonic dependence on protein concentration.

Together, these results indicate that Stx1 clustering does not substantially alter the magnitude of local membrane disorder generated by individual JMD–TMD regions. Instead, local lipid disorder appears to be an intrinsic property of the Stx1 JMD–TMD region that remains largely robust to changes in protein density, palmitoylation state, and clustering behavior.

### 2.8. Clustering Promotes Spatial Overlap of Local Membrane Perturbation Fields

**Figure 7:**
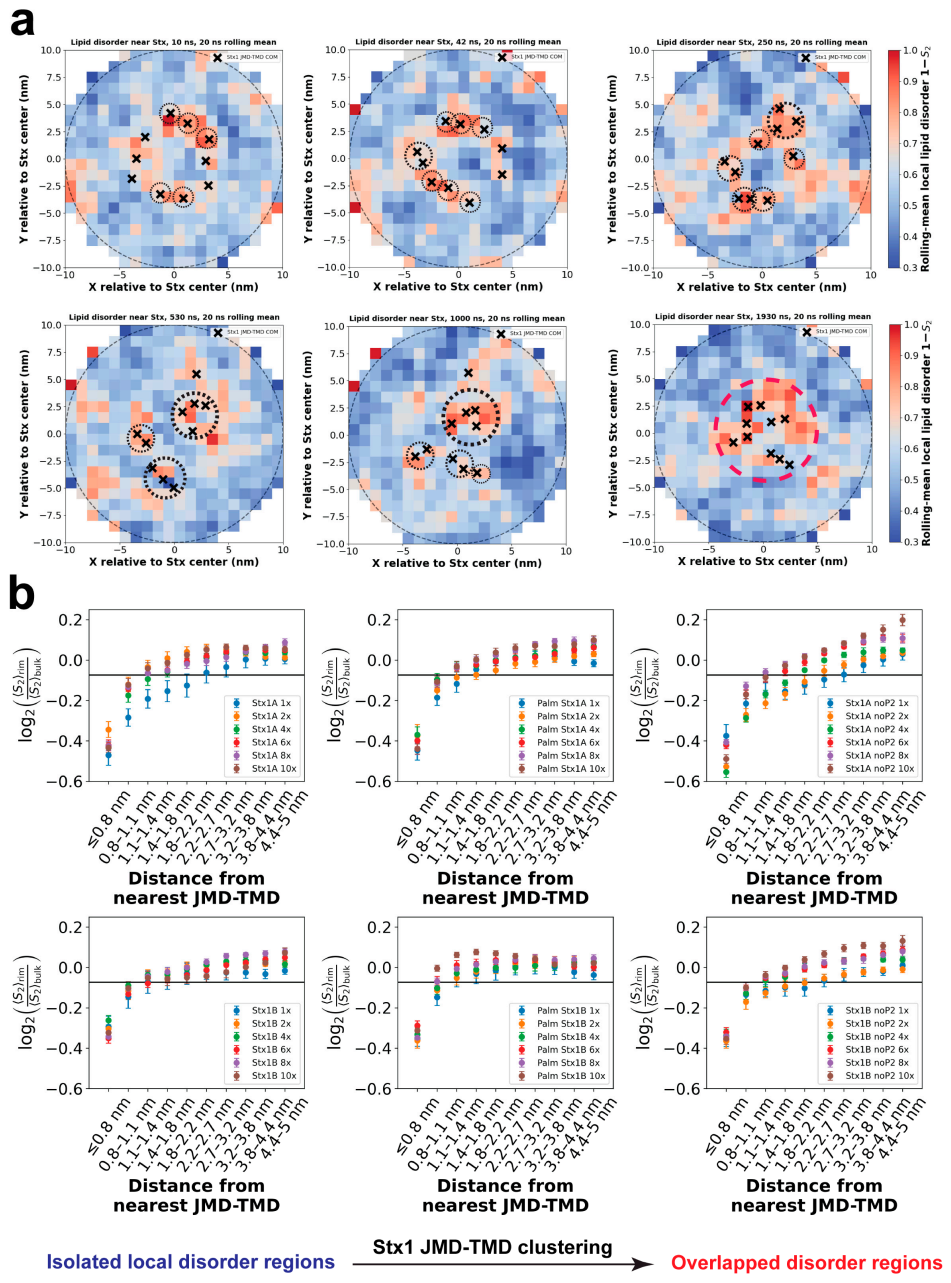
Clustering promotes overlap of local membrane perturbation fields generated by Stx1 JMD–TMD regions. (**a**) Representative lipid-disorder heatmaps from a simulation containing 10 copies of Stx1A JMD–TMD. The snapshots correspond to the frames shown in Figure 3b. Black crosses indicate the center-of-mass positions of individual Stx1A JMD–TMD regions. Dash circles: lipid disorder regions. As Stx1A molecules progressively assemble into oligomeric clusters, their localized membrane perturbation fields increasingly overlap, resulting in the emergence of a confined membrane disorder zone marked by the red dash circle. All heatmaps are displayed using the same color scale. Lipid disorder was calculated as 1 − S_2_ using a 20 ns rolling average (±10 ns around each frame), and only lipids located within 10 nm of the center of mass of the 10 Stx1A JMD–TMD molecules were included. (**b**) Radial profiles of membrane lipid disorder for increasing copy numbers of Stx1A, palmitoylated Stx1A, PIP_2_-depleted Stx1A, Stx1B, palmitoylated Stx1B, and PIP_2_-depleted Stx1B. Increasing protein density progressively shortens the apparent spatial extent of membrane perturbation, consistent with increasing overlap of neighboring perturbation fields rather than an increase in the magnitude of local lipid disorder. Error bars represent SEM. Increasing protein density progressively confines membrane perturbation into a localized disorder zone through spatial overlap of neighboring perturbation fields, rather than through cooperative amplification of membrane disorder around individual Stx molecules. Color codes are for different numbers of copies of Stx JMD-TMD fragments. Black line: Fold change of 95% of the bulk lipid ordering parameters.

Analysis of the spatial distribution of lipid disorder during the clustering process revealed that the localized membrane perturbations generated by individual Stx1A JMD–TMD regions became increasingly concentrated as oligomerization progressed (Figure 7a). As the proteins assembled hierarchically into larger clusters, neighboring regions of elevated lipid disorder increasingly overlapped, producing a confined membrane region with increased overlap of local disorder surrounding the protein assembly.

To quantify this effect, we calculated the $\log_{2}\left(\frac{{\langle S_{2}\rangle }_{rim}}{{\langle S_{2}\rangle }_{bulk}}\right)$ of local lipid order parameters relative to bulk membrane values as a function of radial distance from the nearest Stx1 JMD–TMD region (Figure 7b). Increasing the number of Stx1A copies progressively shortened the apparent radial extent of detectable lipid disorder to approximately 1.1–1.4 nm and converged with the increased protein copies. This observation is consistent with increasing spatial overlap of neighboring perturbation fields and local reorganization of membrane packing within Stx1A clusters. In contrast, increasing the copy number of Stx1B produced little additional reduction in the radial disordering range, consistent with the more localized membrane perturbation generated by individual Stx1B JMD–TMD regions. Similar trends were observed for palmitoylated Stx1 and for Stx1 in PIP_2_-depleted membranes, although the absolute radial ranges differed between conditions.

Together, these observations suggest that Stx1 clustering primarily reorganizes the spatial distribution of membrane disorder rather than increasing the magnitude of local lipid disorder generated by individual JMD–TMD regions. These findings further support the conclusion that local membrane perturbation is predominantly determined by hydrophobic mismatch between the Stx1 transmembrane domain and the surrounding lipid bilayer, whereas clustering concentrates these local perturbations into confined membrane regions without cooperatively enhancing TMD tilting or local lipid disorder.

## 3. Discussion

### 3.1. Local Lipid Disorder Associated with Hydrophobic Mismatch of the Stx1 JMD–TMD Region May Facilitate Stalk Formation During Membrane Fusion

Our simulations indicate that the local lipid disorder surrounding the Stx1 JMD–TMD region is closely associated with hydrophobic mismatch between the Stx1 TMD and the hydrophobic core of the plasma membrane and is further modulated by electrostatic JMD–PIP_2_ interactions. Several observations support this conclusion.

First, the magnitude of local lipid disorder was largely insensitive to Stx1 copy number, clustering state, TMD palmitoylation, PIP_2_ depletion, and mutations that swap C terminal Stx1A/1B TMD sequences. Similarly, these perturbations did not substantially alter the Stx1 TMD tilting order parameter (Figure 1 and Figure 2 and Figure S4). In contrast, Stx1A alone consistently induced stronger local lipid disorder than Stx1B. C terminal swapping mutations (Figure 2a) showed that mutating the Stx1A C-terminal’s last three residues to the last four residues in Stx1B is sufficient to reduce local lipid disorder whereas the reverse mutations from Stx1B back to Stx1A cannot fully restore the higher local lipid disorder (Figure 2c, bottom). Together with the consistent positive rim-wise correlations between local lipid disorder and local interleaflet distance (Figure 1h and Figure 2f,g), these observations indicate that the intrinsic Stx1 TMD sequence establishes the characteristic membrane phenotype, which is closely associated with local hydrophobic mismatch.

How might these local membrane perturbations contribute to membrane fusion? A recent model proposed by the Joseph Rizo laboratory suggests that the JMD–TMD regions of SNARE proteins may function in a detergent-like manner to facilitate exposure of lipid tails and thereby catalyze stalk formation during membrane fusion [24,25]. Such a mechanism would likely be promoted by local lipid disorder, in which lipids deviate from tightly packed conformations aligned parallel to the membrane normal and transiently expose hydrophobic acyl chains to the surrounding environment.

In addition to generating hydrophobic defects, local membrane disorder is also expected to promote membrane deformation. Previous coarse-grained MD simulations demonstrated that clustering of t-SNARE transmembrane domains reorganizes the surrounding membrane into lipid nanodomains enriched in cholesterol and PIP_2_, and induces local membrane curvature, thereby promoting closer membrane apposition [21]. Consistent with this concept, an approximate membrane-curvature analysis [30] of a representative clustering trajectory (Figure S6) qualitatively revealed localized membrane deformation associated with clustered Stx assemblies. Local lipid disorder and membrane deformation are closely linked to complementary consequences of protein-induced membrane perturbation rather than independent phenomena. Such membrane deformations are expected to facilitate the spatial concentration of hydrophobic defects, thereby lowering the energetic barrier for stalk nucleation [31]. In this context, the membrane perturbations induced by clustered Stx molecules may simultaneously enhance local lipid disorder and membrane curvature, together creating a membrane environment favorable for fusion stalk formation. This interpretation is also consistent with the pronounced coalescence of local lipid disorder into a clustered disordered membrane domain observed in Figure 7a, where individual disordered regions merge into a larger disordered membrane domain upon Stx clustering.

Based on our simulations, we propose that clustered Stx molecules at the active zone may collectively generate a locally disordered membrane environment surrounding the SNARE assembly. During vesicle docking and SNARE complex zippering, close membrane apposition between the vesicle and plasma membrane could allow these locally disordered regions to promote hydrophobic tail exposure from both membranes, thereby lowering the energetic barrier for stalk nucleation.

Interestingly, the magnitude of local lipid disorder induced by individual Stx1 JMD–TMD regions remained largely unchanged across different clustering conditions. Increasing Stx copy number may primarily expand the spatial extent of the disordered membrane rim rather than increasing the disordering strength of individual proteins. Such cooperative enlargement of membrane-disordered regions may facilitate formation of fusion intermediates required for membrane mergers.

### 3.2. The Stx1B TMD Sequence Promotes Distinct Membrane Organization and May Facilitate Higher-Order Clustering

Our simulations further show that, compared to Stx1A, Stx1B is less sensitive to TMD palmitoylation, inducing restriction of the spatial extent of membrane perturbation, whereas it exhibits a stronger dependence on membrane PIP_2_ content (Figure 2c). Interestingly, the radial distribution of lipid disorder surrounding palmitoylated Stx1A closely resembles that of non-palmitoylated Stx1B, while palmitoylation produces only modest changes in the radial membrane perturbation profile of Stx1B. These observations suggest that the intrinsic TMD sequence of Stx1B may promote a membrane organization state similar to that induced by TMD palmitoylation in Stx1A. This parallels the experimental observations that Stx1B supports spontaneous release better than Stx1A [11]; however, this deficiency of Stx1A is overcome when Stx1A TMD is palmitoylated [28], although the functional significance of this similarity remains to be established experimentally.

Consistent with these membrane properties, Stx1B also exhibited a modest but reproducible tendency toward larger oligomeric assemblies than Stx1A under intermediate protein-density conditions, particularly in palmitoylated membranes and under PIP_2_ depletion (Figure 5 and Figure S2). Although the difference in mean cluster size between Stx1A and Stx1B under the six-copy condition did not reach statistical significance (Figure S2), the average cluster size of six-copy Stx1B was comparable to that observed for eight-copy Stx1B, suggesting an intrinsic tendency toward higher-order oligomerization.

A plausible structural explanation is that the intrinsic TMD sequence of Stx1B better supports hydrophobic packing interactions that stabilize oligomeric assemblies. The validation simulations demonstrated that introducing the terminal leucine into Stx1A or deleting it from Stx1B produced only modest changes in the radial lipid disorder profiles, indicating that the terminal residue alone is insufficient to account for the distinct membrane-disordering properties of the two isoforms (Figure S1). In contrast, extending the sequence substitutions to include the remaining C-terminal residues produced substantially larger shifts in the membrane phenotype. In the Stx1A background, substitution of the C-terminal Stx1B sequence largely shifted the radial lipid-disorder profile toward that of wild-type Stx1B, whereas the reciprocal substitutions in Stx1B produced only a ~69% recovery (*p* ≈ 0.06) toward the Stx1A phenotype. These results indicate that the C-terminal sequence constitutes the major determinant of the isoform-specific membrane phenotype, whereas the remaining TMD sequence differences contribute cooperatively to fine-tune the magnitude of membrane perturbation. Together with the consistent association between local lipid disorder and hydrophobic mismatch, these observations support a model in which the intrinsic TMD sequence progressively tunes membrane organization rather than being dictated by a single terminal residue. Identifying the relative contributions of individual TMD residues and their cooperative interactions will require additional computational and experimental studies.

Such clustering differences may have important functional implications for membrane fusion. Cooperative assembly of multiple SNARE complexes has long been proposed to enhance force generation during membrane merger [16,32]. In this context, the greater propensity of Stx1B to maintain higher-order oligomeric assemblies may facilitate local concentration and coordination of multiple SNARE complexes within confined membrane regions, thereby promoting cooperative membrane deformation and potentially lowering the energetic barrier for membrane fusion.

Spontaneous neurotransmitter release has been implicated in synaptic maturation, circuit refinement, and neuronal development [33,34]. Although the present simulations do not directly address exocytotic kinetics, the distinct clustering behavior of Stx1B may provide a structural framework for isoform-specific regulation of membrane organization and SNARE assembly. Together, these observations suggest that subtle differences in membrane interactions between Stx1 isoforms may influence the balance between reservoir-like clustering and fusion-competent membrane organization, thereby contributing to functional specialization during neurotransmitter release.

### 3.3. Palmitoylation May Limit Higher-Order Stx1 JMD–TMD Oligomerization and Promote Release from Reservoir-like Assemblies

Previous experimental studies have suggested that Stx proteins can form clustered membrane reservoirs prior to SNARE complex assembly and membrane docking [15,23]. To prevent formation of non-productive SNARE assemblies containing multiple Stx molecules and SNAP25 [35], Munc18 is thought to keep Stx proteins in a closed conformation [2]. A recent study suggests that Munc18 may prevent assembly of Stx oligomers and promote formation of fusion-competent SNARE complexes [15].

Our simulations raise the possibility that palmitoylation may also contribute to regulation of Stx reservoir dynamics. Specifically, palmitoylation suppressed formation of higher-order oligomers for Stx1A, although for Stx1B the mean cluster size was significantly lower only at 10 copies (Figure 5). These observations suggest that palmitoylation disfavors formation or stabilization of large Stx oligomeric assemblies.

Combined with our previous findings that Stx1A palmitoylation promotes upright conformations of the Stx SNARE domain (SND) and facilitates SNARE assembly [36], we propose that palmitoylation may coordinately regulate both membrane organization and SNARE complex availability.

One possible interpretation is that palmitoylation shifts the equilibrium away from large reservoir-like Stx assemblies and toward smaller oligomer states that are more accessible for productive SNARE complex formation. In this model, palmitoylation would potentially promote efficient transition from inactive Stx reservoirs to active SNARE assembly pathways.

Such a mechanism may also help to explain previous observations linking Stx1A TMD palmitoylation to support spontaneous neurotransmitter release [28], although direct experimental validation will be required to establish the relationship between palmitoylation-dependent oligomer regulation and exocytotic activity.

### 3.4. Stx1 JMD–PIP_2_ Interactions Are Closely Associated with Stx1 JMD–TMD Clustering

The newly identified PIP_2_ redistribution provides a mechanistic explanation for the strong dependence of higher-order Stx1 JMD–TMD clustering on membrane PIP_2_ observed in the present study. As Stx1 molecules progressively assembled into larger oligomeric clusters, isolated PIP_2_-enriched regions surrounding individual JMD–TMDs gradually merged into a larger PIP_2_-rich membrane domain (Figure 6a). Quantitative analysis further demonstrated that increasing Stx1 copy number progressively recruited a substantial fraction of IC PIP_2_ molecules into the vicinity of the protein cluster while maintaining an approximately seven-fold local enrichment relative to the bulk membrane (Figure 6b,c). These observations suggest that PIP_2_ is not merely associated with Stx1 clustering but is actively reorganized together with the assembling protein clusters.

The polybasic JMD of Stx contains multiple positively charged residues capable of interacting electrostatically with multivalent PIP_2_ molecules, which carry substantially higher negative charge than monovalent anionic lipids such as phosphatidylserine (PS). Our results suggest that these multivalent electrostatic interactions not only recruit PIP_2_ molecules locally but also progressively concentrate them into a confined membrane region as neighboring Stx clusters merge. Such cooperative lipid redistribution provides a plausible explanation for why depletion of PIP_2_ selectively suppresses higher-order oligomerization while producing only modest effects on the local magnitude of membrane disorder. Although the present simulations do not distinguish between contributions from electrostatic interactions and membrane spontaneous curvature (Figure S6), the preferential recruitment and local enrichment of highly charged PIP_2_ molecules support a model in which multivalent electrostatic interactions play a central role in stabilizing higher-order Stx1 clusters. We therefore propose that hydrophobic mismatch and PIP_2_ recruitment represent two complementary mechanisms regulating Stx1 membrane organization: hydrophobic mismatch primarily determines the generation of local membrane disorder, whereas PIP_2_ redistribution stabilizes and coordinates the assembly of larger Stx1 clusters.

### 3.5. Limitations and Future Directions

Several limitations of the present study should be acknowledged.

First, our simulations employed the MARTINI v2.1 coarse-grained force field to access longer timescales and larger membrane systems without prohibitively increasing computational cost. Although this approach enables efficient exploration of collective membrane organization and protein clustering dynamics, coarse-grained representations inevitably sacrifice chemical resolution and simplify intra- and intermolecular interactions compared with atomistic simulations [5].

Second, the Stx1 JMD was modeled predominantly in a helical conformation based on our previous work. The conformational stability and structural dynamics of the JMD, together with the molecular origin of hydrophobic mismatch-induced membrane perturbation, should be further validated using atomistic molecular dynamics simulations and complementary experimental approaches.

Third, the present study employs isolated JMD–TMD fragments in a coarse-grained membrane model to specifically investigate the membrane interactions mediated by the membrane-proximal region of Stx. Future studies incorporating full-length Stx together with Munc18, SNAP-25, and SNARE complexes, as well as atomistic molecular dynamics simulations, will provide additional insight into how higher-order protein conformations influence membrane organization. Such atomistic simulations will be particularly valuable for characterizing local molecular interactions, whereas coarse-grained simulations remain advantageous for investigating long-timescale lipid redistribution, membrane remodeling, and Stx clustering in complex plasma membranes.

Fourth, the present analysis focused on fundamental descriptors of Stx1 oligomerization, including the fraction of proteins participating in clusters, maximum cluster size, and mean cluster size. More comprehensive characterization of cluster dynamics, including cluster lifetime distributions, cluster area, spatial morphology, fusion and fission events, and temporal evolution of individual oligomers, remains to be investigated. Furthermore, because the simulations were limited to 2 μs, the observed clustering behavior primarily reflects early-stage oligomerization dynamics at the simulated protein densities.

Fifth, the present study focuses on the role of physiological PIP_2_ and dual TMD palmitoylation on Stx clustering and local membrane disorder. Future studies will systematically investigate the concentration dependence of PIP_2_, the contributions of other phosphoinositide species, and possibly the effects of partial TMD palmitoylation to establish quantitative dose–response relationships underlying Stx-mediated membrane organization.

Sixth, the present study focuses on membrane organization at physiological temperature (310 K). Future studies will be needed to investigate temperature-dependent changes in membrane physical properties and their influence on lipid disorder, PIP_2_ redistribution, and Stx clustering.

Future work will therefore focus on multiscale characterization of Stx-mediated membrane organization. Atomistic simulations will be performed to validate JMD secondary structure and TMD-induced membrane perturbation, whereas larger-scale coarse-grained simulations will investigate reservoir-like Stx assemblies and their regulation by palmitoylation and membrane lipid composition. In parallel, machine-learning and deep-learning approaches will be developed to quantify cluster morphology, spatial coverage, lifetime distributions, and dynamic remodeling, providing a more comprehensive description of Stx clustering and its transitions toward fusion-competent membrane organization. Future studies will further investigate how JMD electrostatic interactions and membrane lipid organization regulate the spatial overlap of local membrane perturbation fields within Stx clusters and how these emergent membrane properties contribute to SNARE assembly and early membrane fusion intermediates.

## 4. Materials and Methods

### 4.1. Stx1 Based Models

MARTINI coarse-grained molecular dynamics (CGMD) simulations were performed using 1, 2, 4, 6, 8 or 10 copies of the Stx1A JMD–TMD region (residues 256–288) or Stx1B JMD–TMD region (residues 255–288) embedded in a plasma membrane model with the same lipid composition as described previously [36].

The atomistic reference structure of Stx1A was obtained from our previous study [37]. Initial Stx1B structures were generated by substituting residues that differ from Stx1A (Figure 1a) using PyMOL v2.5.0 [38], thereby preserving identical backbone conformations and equivalent initial geometries across isoforms.

For Stx1B variants containing additional C-terminal residues, the missing residues were appended by aligning the final five residues of the AlphaFold-predicted structure to the Stx1A reference structure in PyMOL v2.5.0.

To evaluate the contribution of the C-terminal sequences, validation simulations were performed. For single-copy Stx1A containing an additional C-terminal leucine (Stx1A L) and single-copy Stx1B lacking the terminal leucine (Stx1B Δ*L*). The reciprocal mutants Stx1A-IFTLL and Stx1B-TLIF were initialized from the WT Stx1B and Stx1-A systems, respectively. All simulation parameters were identical to those used for the corresponding wild-type systems.

### 4.2. CG Model Conversion and MD Simulations

All atomistic Stx models were converted into MARTINI coarse-grained representations using the MARTINI 2.1 force field and a modified version of *martinize_CYP.py* (v2.5), as described previously [36]. The script simultaneously generated the corresponding topology (.itp) files.

Membrane self-assembly and production simulations were performed following protocols described previously [36,39] to generate systems whose box size is ~20 × 20 × 15 nm^3^. For each Stx construct multiple copies of Stx proteins were generated by duplicating and rotationally arranging the proteins around the membrane normal with angular intervals of 360°/*N*, where *N* = 1, 2, 4, 6, 8 or 10.

Plasma membrane systems were self-assembled for each Stx copy number using the same membrane composition described previously [37,39]. The detail compositions are noted in Table S6. For PIP_2_-depleted simulations, all PIP_2_ molecules were replaced with phosphatidylserine (PS). Details of the compositions are noted in Table S7.

For each initial condition, *n* = 10 independent simulations of 2 μs duration were performed to ensure robust conformational sampling.

### 4.3. MD Simulation Parameters

All simulations were performed using GROMACS version 2024 [40] in Bridges-2 high-performing super computer [41].

During the 1 ns equilibration phase, a Berendsen thermostat (310 K) and Berendsen barostat (1 bar) with coupling constants (τ) of 1.0 ps were applied with a timestep of 0.01 ps. Production simulations employed a velocity-rescale thermostat (310 K, τ = 1.0 ps) together with a semi-isotropic Parrinello–Rahman barostat (1 bar, τ = 12.0 ps) with a timestep of 0.02 ps.

The neighbor list was updated every 20 steps using a Verlet-buffer tolerance of 0.005. For each initial condition, *n* = 10 independent simulations with different random seeds were performed for 2 μs. Trajectory frames were saved every 1 ns.

Additional simulation details are provided in the Data Availability section.

### 4.4. Analysis

#### 4.4.1. Trajectory Preprocessing

Following the 2 μs production simulations, all trajectories were preprocessed using GROMACS to reconstruct fragmented molecules across periodic boundary conditions and remove water molecules and ions for subsequent analyses.

#### 4.4.2. Lipid Selection for Binding and Disorder Analysis

Lipids were selected if the minimum distance between any lipid bead and any bead of any Stx protein was within a cutoff distance $r_{crit}$ for a given simulation frame. The total number of selected lipids was defined as $N_{selected}\left(t,\;r_{crit}\right)=\sum_{i=1}^{N_{lipid}}\delta (r_{crit}-r_{i}),$ where δ(x)=1 for x≥0, and δ(x)=0 otherwise. The number of Stx-bound lipids was defined as $N_{bound}\left(t\right)=N_{selected}\left(t,\;r_{crit}=0.8\;nm\right)$.

#### 4.4.3. Stx1 Tilting Ordering Parameters

The orientational order parameter of the Stx1 JMD–TMD region was defined as Equation (2), where $\theta_{TMD,i}\left(t\right)$ is the tilt angle between the vector fit to all of the backbone beads of the TMD residues of the *i*th Stx1 JMD–TMD fragment and the membrane normal at time *t*, and 〈 〉 denotes averaging over all Stx1 molecules in the simulation system. For simulations containing a single Stx1 JMD–TMD molecule, $S_{2,Stx}\left(t\right)$ was calculated directly from the tilt angle of that molecule without ensemble averaging.

#### 4.4.4. Lipid Disorder Parameter and Bulk Lipid Disorder Parameter Calculation

For each selected lipid except cholesterol, the angle $\theta_{i}$ between the vector formed by the second and third lipid beads and the membrane normal (z-axis) was calculated. Each lipid except cholesterol contributes two $\theta_{i}$ values. For cholesterol, $\theta_{i}$ was defined using the vector connecting the R5 and ROH beads.

The lipid disorder parameter $S_{2}$ was defined as Equation (1), where 〈 〉 denotes averaging over all selected lipids $r_{crit}$ at time t.

The bulk lipid order parameter was defined as the lipid order parameter calculated using all membrane lipids, corresponding to the limiting case in which the cutoff distance approaches infinity:

(4) $$S_{2}^{bulk}\left(t\right)=\underset{r_{\mathrm{crit}}\to +\infty }{\lim}S_{2}\left(t,\;r_{crit}\right)$$

For each simulation, $S_{2}\left(t,\;r_{crit}\right)$ was calculated at $r_{crit}=$ 0.8, 1.1, 1.4, 1.8, 2.2, 2.7, 3.2, 3.8, 4.4, and 5 nm.

The lipid disorder parameter within each annular rim was calculated by selecting lipid residues whose minimum distance between any lipid bead and any bead of any Stx molecule was (≤0.8, 0.8–1.1, 1.1–1.4, 1.4–1.8, 1.8–2.2, 2.2–2.7, 2.7–3.2, 3.2–3.8, 3.8–4.4, and 4.4–5.0 nm).

#### 4.4.5. Local Hydrophobic Mismatch Analysis

Local membrane hydrophobic mismatch was approximated from the local interleaflet headgroup distance surrounding the Stx1 JMD–TMD region compared that of the bulk membrane. Lipid molecules neighboring Stx1 were first identified using the same distance-based selection procedure described above. For radial analyses, neighboring lipids were further divided into successive annular rims according to their minimum distance from the Stx1 JMD–TMD region (≤0.8, 0.8–1.1, 1.1–1.4, 1.4–1.8, 1.8–2.2, 2.2–2.7, 2.7–3.2, 3.2–3.8, 3.8–4.4, and 4.4–5.0 nm). Dynamic leaflet assignment and local interleaflet distance calculations are described in Supporting Method. Within each annular rim, the average headgroup positions of the upper (IC) and lower (EC) membrane leaflets were determined, and the local interleaflet distance ($d_{leaflet,rim}$) was calculated as the difference between the mean z-coordinates of the two leaflets. The local hydrophobic mismatch parameter $\Delta L_{rim}$ was then defined as

(5) $$\Delta L_{rim}=d_{leaflet,bulk}-d_{leaflet,rim},$$

where $d_{leaflet,bulk}$ denotes the average interleaflet distance measured within the outermost annular rim (4.4–5.0 nm), which served as the bulk membrane reference. Positive values of ΔL therefore indicate local membrane thinning relative to the bulk membrane. For comparisons with the radial lipid-order analysis, the relative interleaflet distance was additionally expressed as ${log}_{2}\left(\frac{{\langle d_{leaflet}\rangle }_{rim}}{{\langle d_{leaflet}\rangle }_{bulk}}\right),$ analogous to the radial lipid-order analysis.

#### 4.4.6. Rim-Wise Correlation Analysis

To quantify the relationship between local membrane disorder and local hydrophobic mismatch, rim-wise correlation analyses were performed independently for each simulation. For each annular rim, the local lipid order parameter and local interleaflet distance were first averaged over the final 100 ns of the trajectory. Pearson correlation coefficients were then calculated across the corresponding annular rims within each independent simulation. The resulting correlation coefficients from the ten independent simulations were summarized using Fisher’s z-transformation, and the mean correlation together with its 95% confidence interval was reported. Statistical significance was assessed using a one-sample t-test of the Fisher-transformed correlation coefficients against zero.

#### 4.4.7. Stx JMD-PIP_2_ Recruitment Analysis

For simulations containing Stx1A, Stx1B, Palm Stx1A, and Palm Stx1B, the recruitment of PIP_2_ molecules by the Stx1 JMD fragment was quantified using the final 100 ns of each trajectory. Lipid molecules were considered bound if any bead of the lipid was located within 0.8 nm of any bead in the Stx1 JMD region (Figure 1a). Only PIP_2_ molecules located in the IC leaflet were included in the analysis. For this analysis, the IC and EC leaflets were identified in each frame by comparing the z-coordinate of the lipid reference bead (PO4, GM1, or ROH) with the instantaneous membrane midplane, defined as the average z-coordinate of all membrane lipid reference beads. Unlike the local hydrophobic mismatch analysis, which required frame-wise local leaflet assignment within each annular rim, the present analysis only required classification of lipids into the two membrane leaflets. Therefore, the instantaneous global membrane midplane provided a sufficient criterion for leaflet assignment.

The fraction of recruited PIP_2_ molecules was calculated as

(6) $$f_{PIP2-Stx}=\frac{N_{PIP2-Stx,IC}}{N_{PIP2,IC}},$$

where $N_{PIP2-Stx,IC}$ is the number of unique intracellular-leaflet PIP_2_ molecules bound to at least one Stx1 JMD region, and $N_{PIP2,IC}$ is the total number of intracellular-leaflet PIP_2_ molecules.

#### 4.4.8. Stx Pairwise Distance Calculation and Multiple Cluster Definition

For simulations containing multiple Stx proteins, pairwise distances were calculated for every protein pair at each simulation frame. The pairwise distance was defined as the minimum contact distance between any backbone (BB) beads of two Stx proteins.

For each trajectory, pairwise distances were stored as matrices with dimensions: (n_frames, n_stx, n_stx).

A Stx cluster was defined as a group containing more than one Stx protein with pairwise distances ≤ 1.0 nm. For each frame, the following quantities were calculated: (1) the total number of clustered Stx proteins $N_{stx,C}\left(t\right)$, finally yielding the fraction of Stx formed cluster $f_{stx,C}\left(t\right)=N_{stx,C}\left(t\right)/N_{stx}$; (2) the maximum number of Stx proteins in a cluster $N_{stx,C}^{max}\left(t\right)$; and (3) the average number of Stx proteins in a cluster $\langle N_{stx,C}\left(t\right)\rangle =\sum_{i=1}^{n_{stx}}i\times N_{i}\left(t\right)/\sum_{i=1}^{n_{stx}}N_{i}\left(t\right)$, where $N_{i}\left(t\right)$ is the number of clusters made of *i* Stx JMD-TMDs at time t. Number of monomers $N_{1}\left(t\right)$ was included.

#### 4.4.9. Statistics

All quantities are reported as mean ± SEM together with the corresponding sample size (N). Averaging was performed over trajectory frames collected after 1900 ns, during which no substantial temporal drift was observed. All error bars are shown as SEM.

Individual data points represent values obtained from each of the *n* = 10 independent simulations.

Statistical comparisons between pairs of groups were performed using the Mann–Whitney U test in SciPy v1.10.1 package (https://docs.scipy.org/doc/scipy-1.10.1/, accessed on 24 July 2026), and the corresponding *p* values are reported in the figures. Comparison of multiple groups together were performed using Kruskal test in SciPy v1.10.1 package and the *p* values are reported in figure captions. Pearson correlations were calculated by comparing quantities with number of Stx in simulations, and *p*-value from statistical significance of the correlations were also calculated using *pearsonr* function from SciPy v1.10.1 package.

#### 4.4.10. Analysis Software

Trajectory analyses were performed in Python v3.8.10 using MDTraj v1.10.0 [42]. Statistical analyses were conducted using SciPy v1.10.1. Figures were generated using Matplotlib v3.7.5, Seaborn v0.11.2, and statannotations v0.6.0. For more information, please visit data availability section.

## 5. Conclusions

In summary, our MARTINI coarse-grained molecular dynamics simulations demonstrate that local membrane disorder induced by the Stx1 JMD–TMD region is primarily associated with hydrophobic mismatch and remains largely independent of Stx clustering state. Although palmitoylation does not substantially alter the magnitude of local lipid disorder, it spatially restricts the radial extent of membrane perturbation, producing a more localized membrane organization that resembles that of Stx1B. In contrast, PIP_2_ depletion broadens the disorder field while simultaneously suppressing higher-order oligomerization, indicating distinct roles of lipid modification and membrane electrostatics in regulating Stx membrane organization.

Our simulations further show that PIP_2_ depletion and TMD palmitoylation suppresses the formation of higher-order oligomers, potentially shifting Stx assemblies toward smaller, fusion-competent states. Stx1B exhibits a modestly greater propensity for oligomerization than Stx1A, consistent with isoform-specific differences in membrane organization and clustering behavior.

Together, these findings support a unified model in which the intrinsic Stx1 TMD sequence establishes an isoform-specific membrane phenotype that is closely associated with local hydrophobic mismatch, with reciprocal sequence substitutions further demonstrating that the C-terminal TMD sequence serves as a major determinant of this membrane organization, whereas palmitoylation and PIP_2_ interactions spatially tune membrane organization and clustering dynamics. Consequently, these mechanisms regulate the transition of Stx assemblies from reservoir-like clusters toward membrane environments permissive for SNARE complex assembly and membrane fusion.

## Data Availability

All files required to reproduce the simulations and analyses described in this study, including starting structures, topology files, molecular dynamics parameter files, processed datasets, and analysis scripts, will be made publicly available upon publication at https://zenodo.org/records/21511612 (accessed on 22 July 2026). The trajectory (.xtc) files are too large to be deposited in the public repository but can be obtained from the corresponding author upon reasonable request.

## Supplementary Materials

The following supporting information can be downloaded at: https://www.mdpi.com/article/10.3390/ijms27156673/s1.

## Abbreviations

The following abbreviations are used in this manuscript:

CG	Coarse grain
MD	Molecular dynamics
Stx	Syntaxin
JMD	Juxtamembane domain
TMD	Transmembrane domain
SND	SNARE domain
Palm Stx1	Palmitoylated Stx1
PIP_2_	phosphatidylinositol 4,5-bisphosphate
noP2	PIP_2_ depletion
IC	intracellular
EC	extracellular
