# Supplementary material for: Lipid Disorder and PIP2-Regulated Clustering of Syntaxin-1 JMD–TMD Regions Govern Membrane Fusion Competence
Source: Int J Mol Sci. 2026 Jul 27;27(15):6673. doi: 10.3390/ijms27156673 (PMC13467315; doi:10.3390/ijms27156673)
Supplement: Supplementary file 1 [file ijms-27-06673-s001.zip › Stx1 lipid disorder SI.pdf]

Supporting information

Supporting Methods

Dynamic leaflet assignment for local interleaflet distance calculation

Within each annular rim surrounding a Stx TMD and at each trajectory frame, neighboring lipid molecules were assigned to the intracellular (IC) and extracellular (EC) leaflets based on the *z*-coordinates of their reference beads. A single reference bead was selected to represent each lipid molecule (PO_4 for phospholipids, GM1 for ganglioside GM1, and ROH for cholesterol). The *z*-coordinates of all reference beads within an annular rim were partitioned into two groups using one-dimensional *k*-means clustering. The cluster with the higher mean *z*-coordinate was assigned to the upper (intracellular, IC) leaflet, whereas the cluster with the lower mean *z*-coordinate was assigned to the lower (extracellular, EC) leaflet.

The local bilayer midplane was defined as the midpoint between the two cluster centers. To minimize ambiguous leaflet assignment, reference beads located within 0.8 nm of the inferred midplane were excluded from subsequent analysis. This procedure primarily removed cholesterol molecules adopting approximately horizontal orientations near the bilayer center, while also excluding any other reference beads whose leaflet identity could not be robustly assigned. Within each leaflet, remaining *z*-coordinate outliers were further removed using a median absolute deviation (MAD)-based criterion with a robust-*z* threshold of 3.5.

The mean headgroup positions of the IC and EC leaflets were then calculated, and the local interleaflet distance for each annular rim was defined as

$$d_{\mathrm{leaflet,rim}} = \langle z_{\mathrm{IC,rim}} \rangle - \langle z_{\mathrm{EC,rim}} \rangle,$$

where $\langle z_{\mathrm{IC,rim}} \rangle$and $\langle z_{\mathrm{EC,rim}} \rangle$denote the mean *z*-coordinates of the reference beads assigned to the IC and EC leaflets, respectively.

The same leaflet-assignment and filtering procedure was applied to the outermost annular rim (4.4–5.0 nm), which served as the bulk membrane reference for hydrophobic mismatch calculations described in the main text.

Supporting Tables

Table S1. Local lipid order parameters, bulk membrane lipid order parameters, and Stx1 TMD tilting order parameters for all Stx1 constructs.

Table S2. Radial analyses of lipid order parameters and hydrophobic mismatch as a function of distance from the Stx1 JMD–TMD region for all Stx1 constructs.

Table S3. log2 fold changes of local lipid order parameters and hydrophobic mismatch relative to bulk membrane values as a function of distance from the Stx1 JMD–TMD region for all Stx1 constructs.

Table S4. Average numbers of Stx1 clusters and fractions of Stx1 JMD–TMD molecules participating in clusters for all Stx1 constructs.

Table S5. PIP_2 recruitment fractions and local PIP_2 enrichment factors of Stx1 JMD–TMD regions for all Stx1 constructs.

Table S6. Average lipid composition and leaflet partitioning of the physiological plasma membrane used in the simulations.

Table S7. Average lipid composition and leaflet partitioning of the PIP_2-depleted plasma membrane used in the simulations.

Note: Supplementary tables S1-S5 are provided as .xlsx files containing mean ± SEM values. For all Stx1 constructs, *n* = 10 independent simulations were analyzed. For table S3, quantity log2(<S_2>_rim/<S_2>_bulk) refers to $\log_2\left(\frac{\langle S_2\rangle_{\mathrm{rim}}}{\langle S_2\rangle_{\mathrm{bulk}}}\right)$ shown in Figure 1e, 2d, 2e, 7b and S1b, and quantity log2(<d_leaflet>_rim/ <d_leaflet>_bulk) refers to $\log_2\left(\frac{\langle d_{\mathrm{leaflet}}\rangle_{\mathrm{rim}}}{\langle d_{leaflet}\rangle_{\mathrm{bulk}}}\right)$ shown in Figure 1g, 2d, 2e and S1b. For table S6-7, values were averaged over independently assembled membrane systems (*n* = 24 for Table S6 and *n* = 12 for Table S7) and are reported as mean ± SD.

Supporting Videos

Video S1. Representative clustering dynamics of 10 copies of Stx1A JMD–TMD embedded in a ~20 × 20 nm^2 plasma membrane, corresponding to the simulation shown in Figure 3b–c. The trajectory was selected from one of ten independent simulations. To facilitate visualization of cluster formation and reorganization, one of the Stx1A JMD-TMD was centered while maintaining the original periodic boundary conditions. PO_4 beads of PIP_2 molecules were colored by blue-violet.

Video S2. Representative clustering dynamics of 10 copies of Palm Stx1A JMD–TMD embedded in a ~20 × 20 nm^2 plasma membrane, corresponding to the simulation shown in the top panel of Figure 6a. PO_4 beads of PIP_2 molecules were colored by blue-violet.

Video S3. Representative clustering dynamics of 10 copies of Stx1A JMD–TMD embedded in a ~20 × 20 nm^2 PIP_2 depleted plasma membrane, corresponding to the simulation shown in the bottom panel of Figure 6a.

Supporting Figures

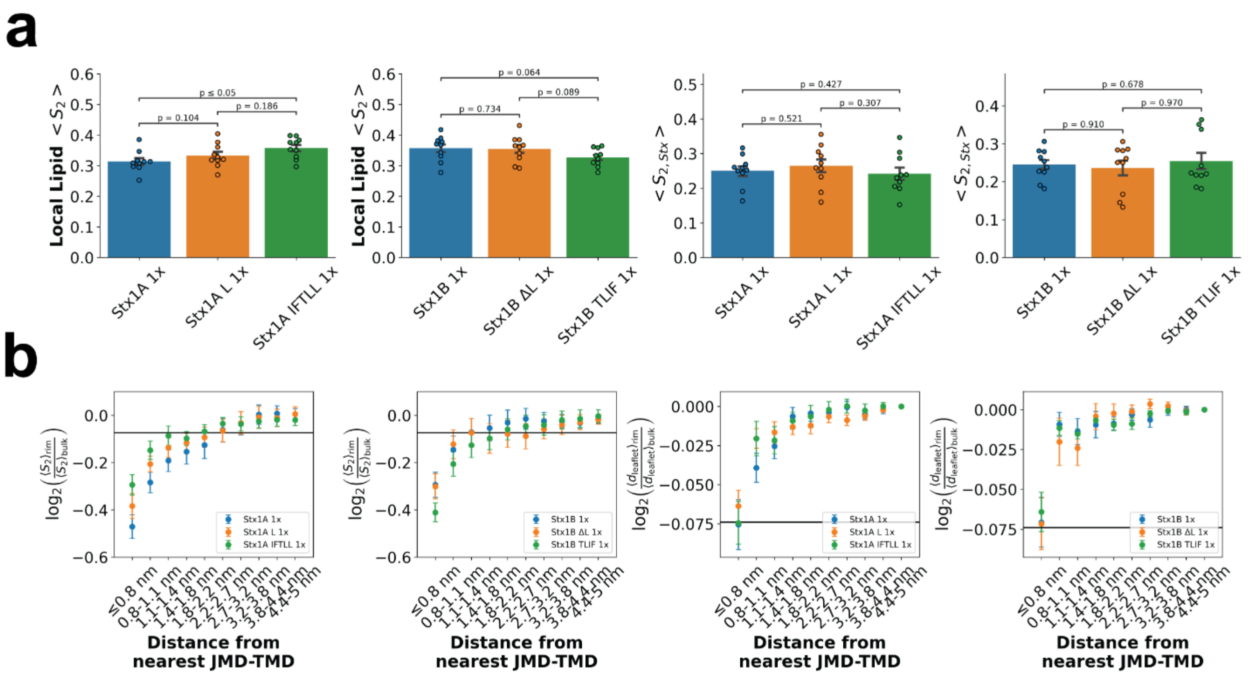

Figure S1. Validation of the contribution of the C-terminal transmembrane sequence to local membrane disorder and hydrophobic mismatch. (a) Comparison of the final 100 ns averages of the local lipid order parameter and local Stx tilting disorder ($\langle S_{2,Stx} \rangle$) for Stx1A and Stx1B together with representative C-terminal TMD mutants. Stx1A mutants include the additional C-terminal leucine mutant (Stx1A L) and the C-terminal TMD-swapped mutant (Stx1A IFTLL). Stx1B mutants include the L288-truncated mutant (Stx1B ΔL) and the reciprocal C-terminal TMD-swapped mutant (Stx1B TLIF) (Figure 2a). Individual dots represent independent simulations. Error bars indicate mean ± SEM. P values were calculated using the Mann–Whitney U test. **(b)** Radial profiles of the relative lipid order parameter, $\log_2\left(\frac{\langle S_2 \rangle_{\text{rim}}}{\langle S_2 \rangle_{\text{bulk}}}\right)$, and the relative interleaflet distance, $\log_2\left(\frac{\langle d_{\text{leaflet}} \rangle_{\text{rim}}}{\langle d_{\text{leaflet}} \rangle_{\text{bulk}}}\right)$, for the corresponding mutants. Values were calculated using the direct annular-rim analysis described in the Methods from the final 100 ns of each simulation.

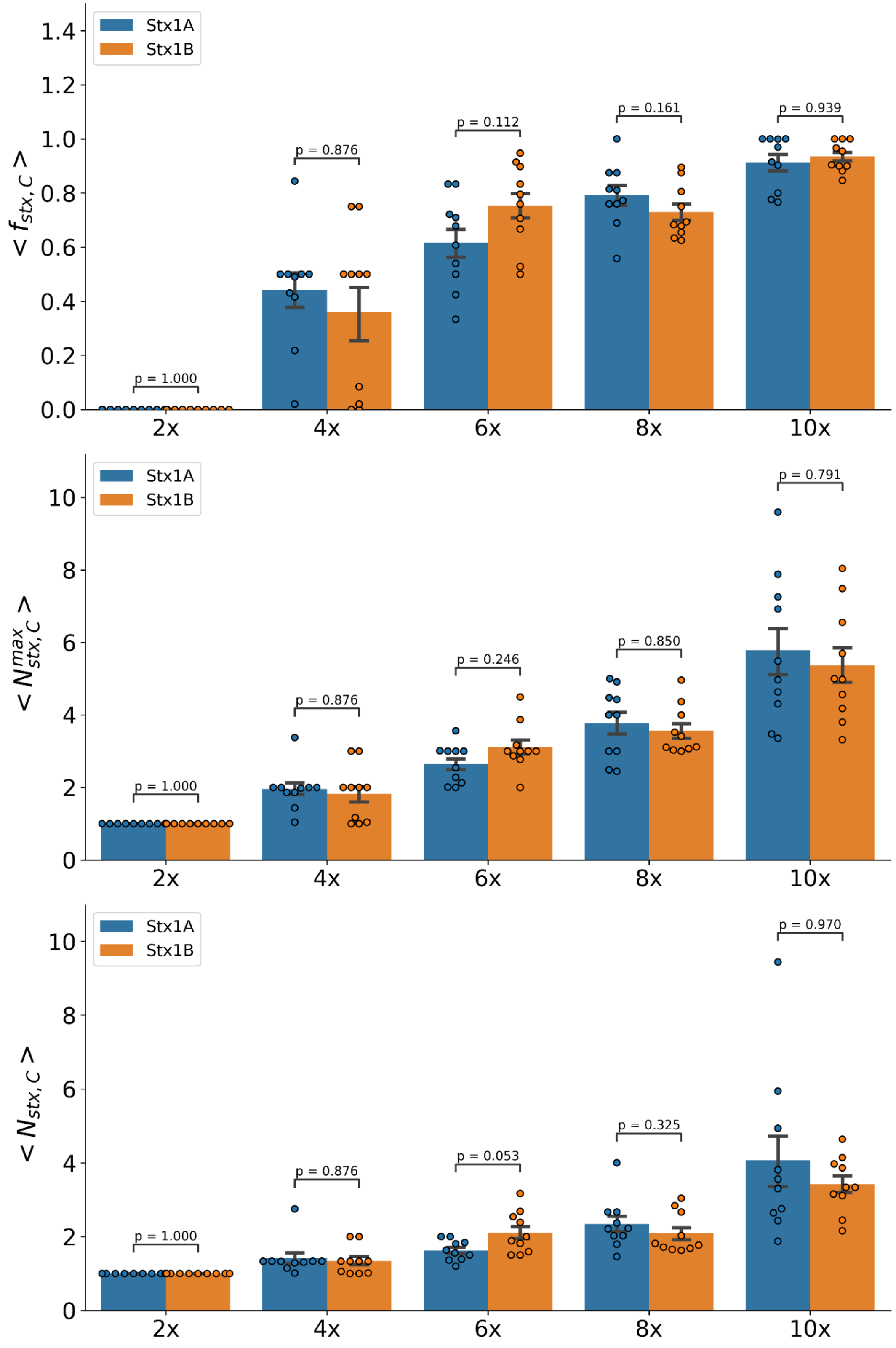

Figure S2. Direct comparison of clustering properties between Stx1A and Stx1B at different protein copy numbers. Final average fraction of Stx1 molecules participating in clusters (top),

maximum cluster size (middle), and mean cluster size (bottom) calculated from the final 100 ns of $n = 10$ simulations. Individual dots represent independent simulations, bars indicate mean ± SEM, and p values were calculated using the Mann–Whitney U test. No statistically significant differences were detected between Stx1A and Stx1B at any protein copy number. Statistical details are provided in Table S5.

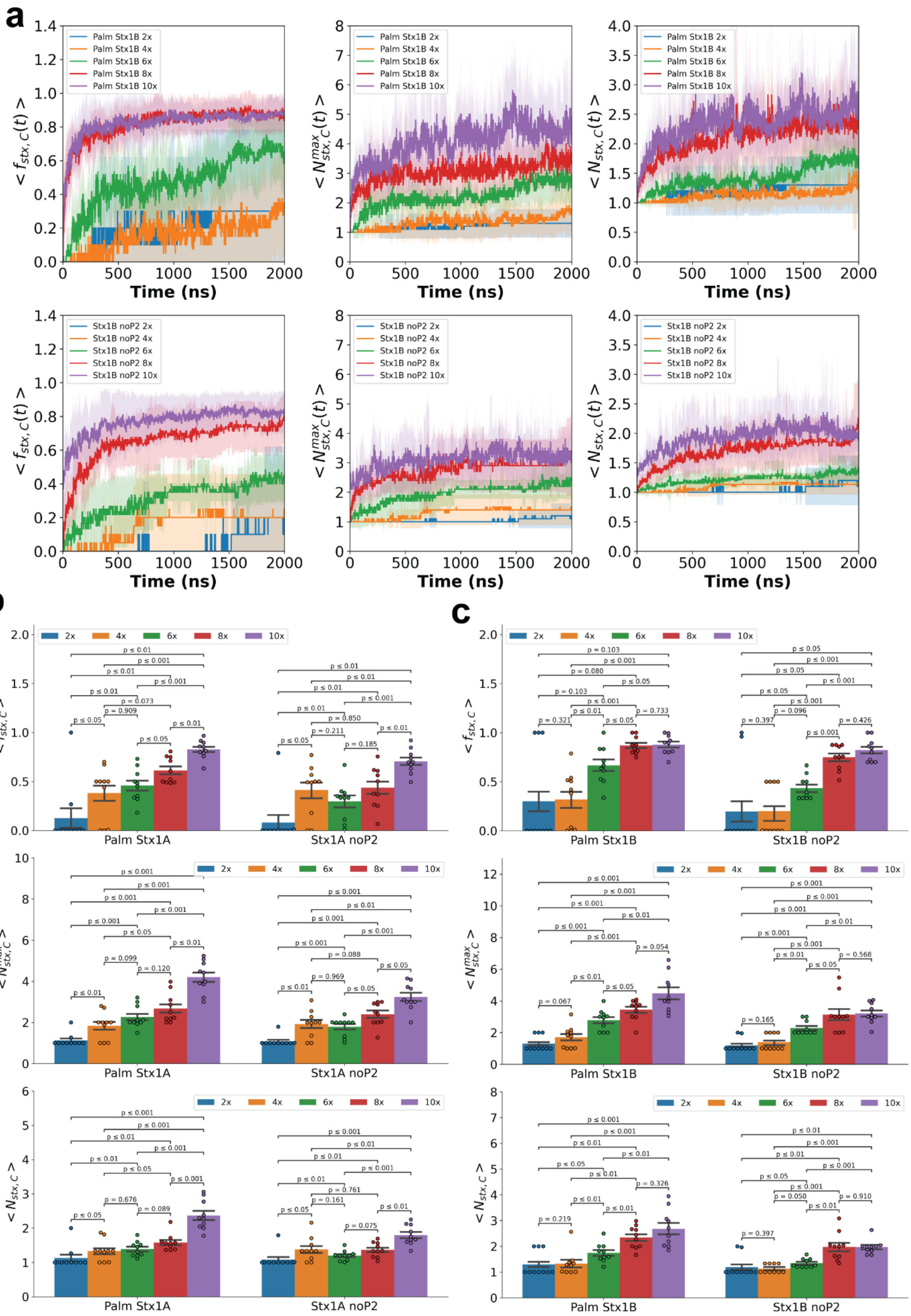

a
Palm Stx1B 2x
Palm Stx1B 4x
Palm Stx1B 6x
Palm Stx1B 8x
Palm Stx1B 10x
Stx1B noP2 2x
Stx1B noP2 4x
Stx1B noP2 6x
Stx1B noP2 8x
Stx1B noP2 10x
$< f_{stx,C}(t) >$
$< N^{max}_{stx,C}(t) >$
$< N_{stx,C}(t) >$
Time (ns)
b
c
2x
4x
6x
8x
10x
$< f_{stx,C} >$
$< N^{max}_{stx,C} >$
$< N_{stx,C} >$
Palm Stx1A
Stx1A noP2
Palm Stx1B
Stx1B noP2

Figure S3. Protein-concentration dependence of Stx1 clustering under palmitoylation and PIP2 depletion. (a) Average trajectories from *n* = 10 simulations showing the fraction of Stx1 JMD–TMD molecules participating in clusters $\langle f_{stx,C}(t)\rangle$, the maximum cluster size $\langle N^{max}_{stx,C}(t)\rangle$, and the mean cluster size $\langle N_{stx,C}(t)\rangle$, for systems containing 2, 4, 6, 8, and 10 copies of palmitoylated Stx1B (Palm Stx1B) or Stx1B in PIP2-depleted membranes (Stx1B noP2). Shaded regions indicate mean ± SD. **(b, c)** Alternative presentation of the clustering data shown in Figure 6c emphasizing protein-concentration dependence. Final values of the fraction of Stx1 molecules participating in clusters, maximum cluster size, and mean cluster size were calculated from the final 100 ns of *n* = 10 simulations for Palm Stx1A and Stx1A noP2 (**b**) and for Palm Stx1B and Stx1B noP2 (**c**). Individual dots represent independent simulations, and error bars indicate mean ± SEM. P values were calculated using the Mann–Whitney U test. Corresponding statistical analyses are provided in Table S5.

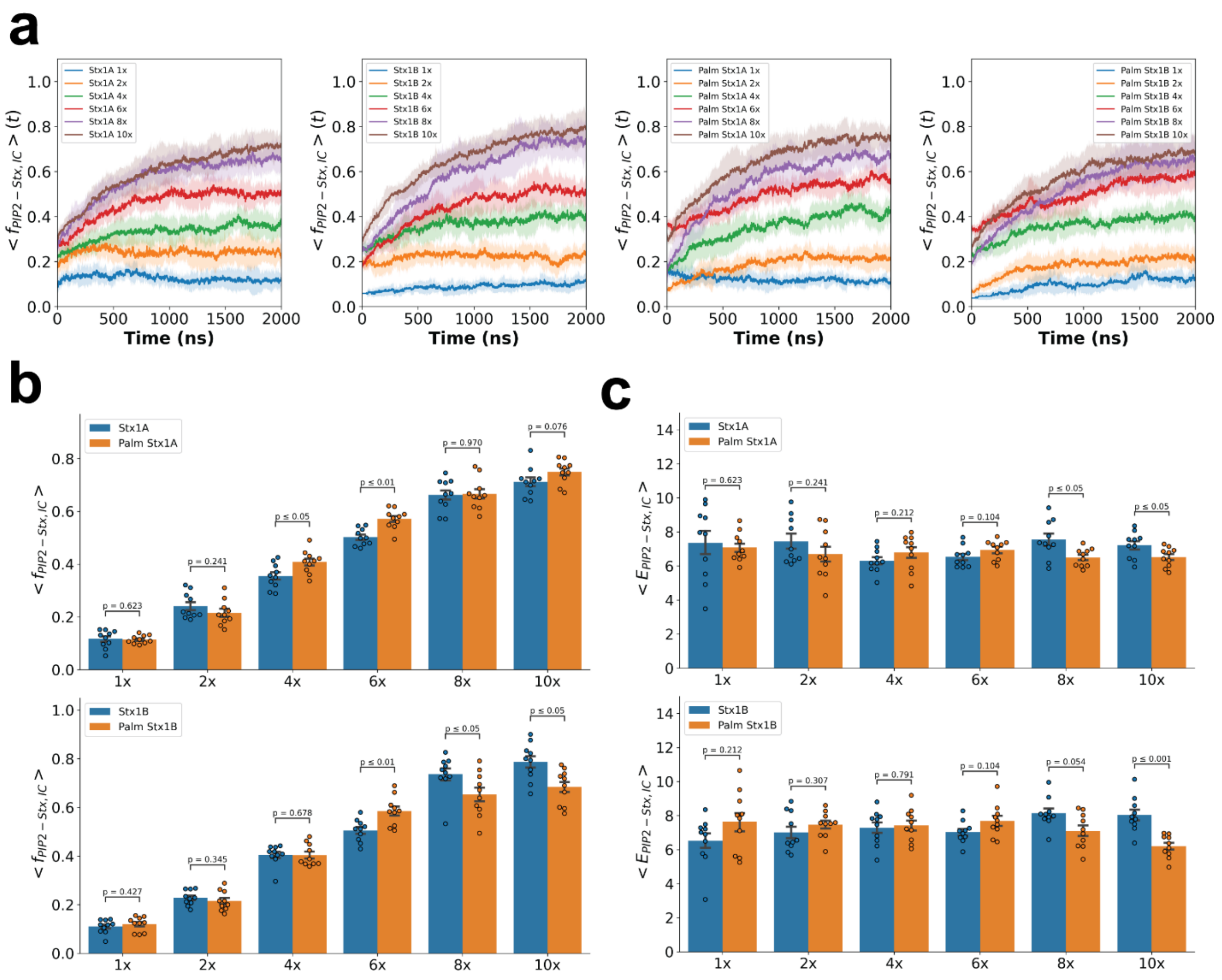

Figure S4. Palmitoylation modulates PIP_2 recruitment and local PIP_2 enrichment in an isoform- and protein copy number-dependent manner. **(a)** Average trajectories of the fraction of intracellular (IC) leaflet PIP_2 molecules bound by at least one Stx1 JMD–TMD region for Stx1A, Stx1B, fully palmitoylated Stx1A (Palm Stx1A), and fully palmitoylated Stx1B (Palm Stx1B) systems containing 1, 2, 4, 6, 8, or 10 copies of the Stx1 JMD–TMD region. PIP_2 recruitment increased progressively with protein copy number and approached a stable plateau during the final 100 ns used for quantitative analysis in panel (b). Shaded regions indicate mean ± SD from n = 10 independent simulations. **(b)** Final fraction of intracellular (IC) leaflet PIP_2 molecules bound by at least one Stx1 JMD–TMD region for non-palmitoylated (Stx1A and Stx1B) and fully palmitoylated (Palm Stx1A and Palm Stx1B) systems containing 1, 2, 4, 6, 8, or 10 copies of the Stx1 JMD–TMD region. Values were averaged over the final 100 ns of n = 10 independent simulations. Palmitoylation produced distinct effects on PIP_2 recruitment depending on both the Stx isoform and protein copy number. While palmitoylation modestly increased PIP_2 recruitment for Stx1A at intermediate copy numbers, it reduced PIP_2 recruitment for Stx1B at higher protein copy numbers. **(c)** Final local PIP_2 enrichment surrounding the corresponding Stx1 JMD–TMD regions, calculated as the ratio between the local PIP_2 fraction within the JMD–TMD-associated membrane region and the average PIP_2 fraction in the intracellular

leaflet. Similar to PIP_2 recruitment, the effect of palmitoylation on local PIP_2 enrichment depended on both Stx isoform and protein copy number. Individual dots represent independent simulations, and bars indicate the mean ± SEM. P values were calculated using the Mann–Whitney U test.

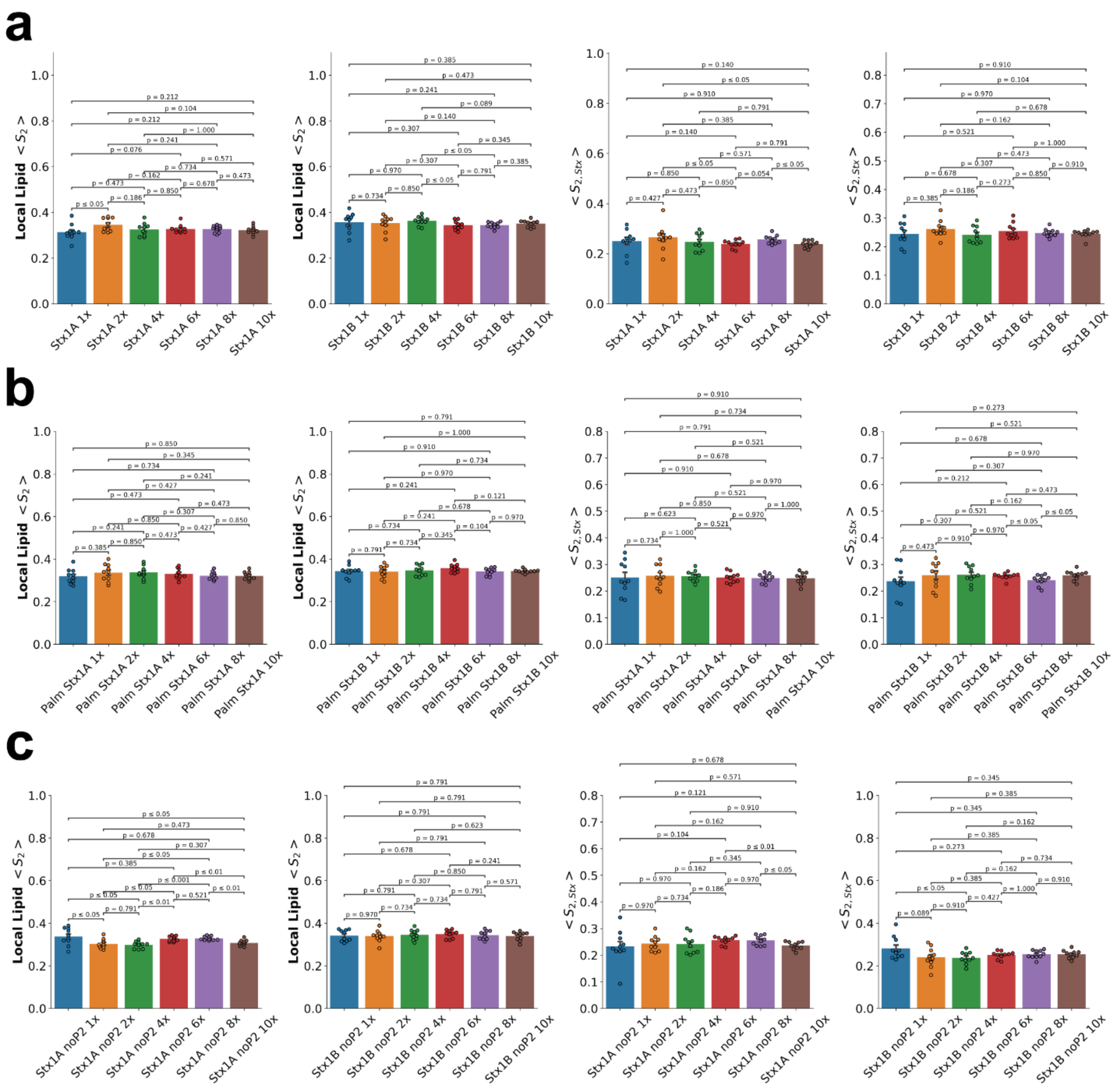

Figure S5. Increasing Stx1 copy number minimally alters local lipid disorder and TMD orientation under physiological, palmitoylated, and PIP_2-depleted membrane conditions. (a) Final local lipid disorder (left two panels) and TMD tilting order parameters (right two panels) for non-palmitoylated Stx1A and Stx1B systems containing 1, 2, 4, 6, 8, or 10 copies of the Stx1 JMD–TMD region. (b) Corresponding analyses for fully palmitoylated Stx1A (Palm Stx1A) and Stx1B (Palm Stx1B). (c) Corresponding analyses for Stx1A and Stx1B in PIP_2-depleted membranes (noP2).

For each system, values were calculated from the final 100 ns of n = 10 independent simulations. Across all three membrane conditions, increasing Stx1 copy number produced only modest changes in local lipid disorder and TMD tilting, indicating that the membrane perturbation generated by individual JMD–TMD regions is largely independent of protein concentration. Individual dots

represent independent simulations, and bars show the mean ± SEM. P values were calculated using the Mann–Whitney U test. Detailed statistical results are provided in Table S1.

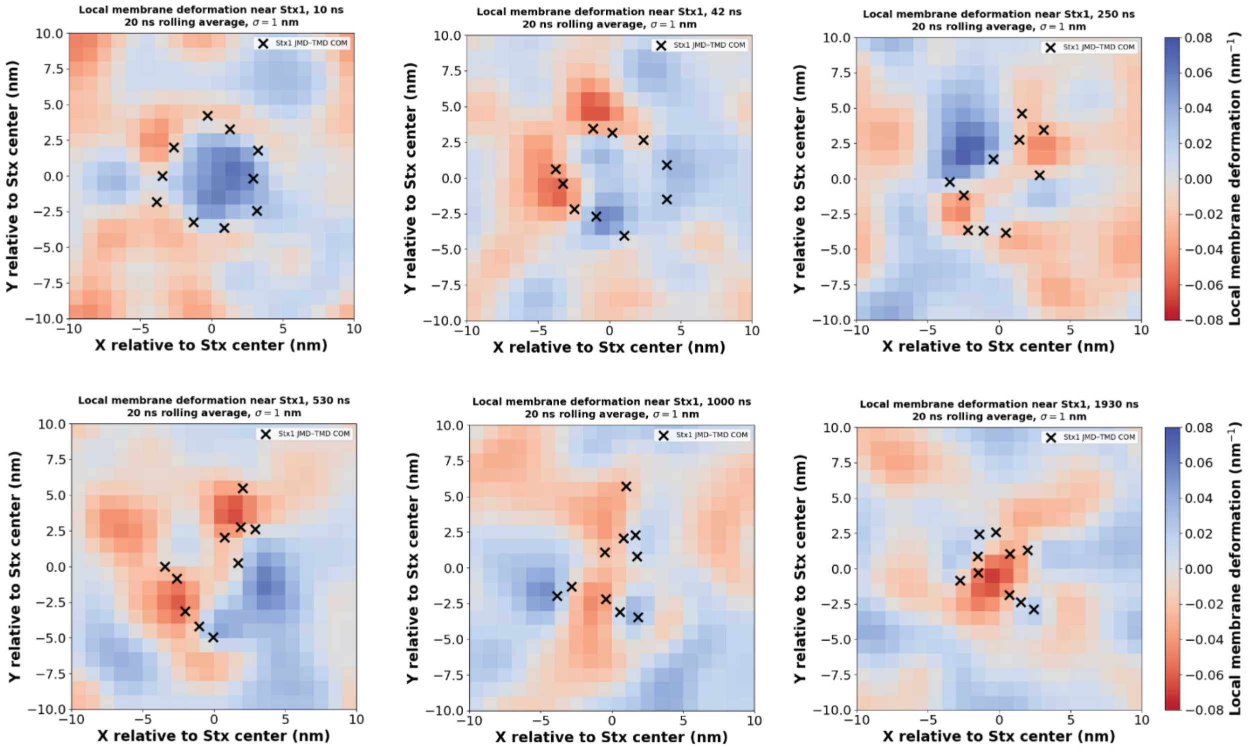

Figure S6. An approximate local membrane deformation proxy was estimated from the Laplacian of a Gaussian-smoothed membrane-height field. The membrane height, $h(x, y)$, was defined as the local average z-coordinate of lipid tail beads using the Monge gauge representation of a continuous membrane surface [1]. To suppress molecular-scale fluctuations and obtain a continuous membrane representation, the membrane-height field was averaged using a 20 ns rolling window (±10 ns around each frame) followed by Gaussian smoothing ($\sigma = 1$ nm). Under the small-slope approximation, the local deformation proxy was calculated as $-\frac{1}{2}\nabla^2 h$. This quantity provides an approximate measure of local membrane deformation rather than a direct calculation of membrane curvature.

References

1. Deserno, M., Fluid lipid membranes–a primer. *http://www.cmu.edu/biolphys/deserno/pdf/membrane_theory.pdf* **2007**,
